# Portable and Autonomous PEDOT-Modified Flexible Paper-Based Methanol Fuel Cell Sensing Platform Applied to L1CAM Recombinant Protein Detection

**DOI:** 10.1021/acsapm.3c02781

**Published:** 2024-03-11

**Authors:** Liliana P. T. Carneiro, Alexandra M. F. R. Pinto, Dzmitry Ivanou, Adélio M. Mendes, M. Goreti F. Sales

**Affiliations:** † BioMark, Sensor Research@UC/CEB, Department of Chemical Engineering, Faculty of Sciences and Technology, University of Coimbra, Rua Sílvio Lima, Pólo II, Coimbra 3030-790, Portugal; ‡ BioMark, Sensor Research@ISEP/CEB, School of Engineering, Polytechnic Institute of Porto, Rua Dr. António Bernardino de Almeida, 431, Porto 4249-015, Portugal; § CEFT, Transport Phenomena Research Center, Department of Chemical Engineering, Faculty of Engineering, University of Porto, Rua Dr. Roberto Frias, Porto 4200-465, Portugal; ∥ AliCE - Associate Laboratory in Chemical Engineering, Faculty of Engineering, University of Porto, Rua Dr. Roberto Frias, Porto 4200-465, Portugal; ⊥ LEPABE - Laboratory for Process Engineering, Environment, Biotechnology and Energy, Faculty of Engineering, University of Porto, Rua Dr. Roberto Frias, Porto 4200-465, Portugal

**Keywords:** PEDOT, paper-based methanol fuel cell, molecularly imprinted polymer, biosensor, L1CAM, electrochromic cell, self-powered, self-signaled

## Abstract

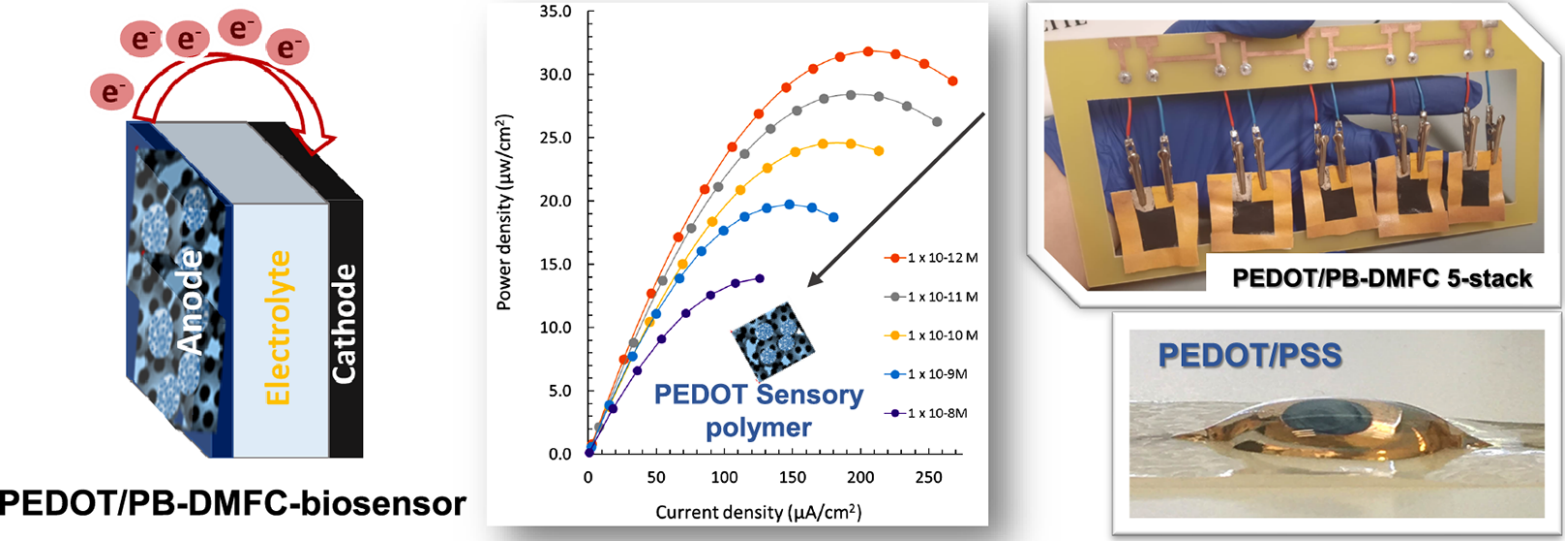

This work describes first a 5-stack direct methanol fuel cell (DMFC) based on poly(3,4-ethylenedioxythiophene)-modified paper (PEDOT/PB-DMFC), which acts as an energy source and biosensor, coupled to an electrochromic cell (EC). It is autonomous and monitors the biosensor response by color change, as appropriate for point-of-care (POC) applications. In detail, DMFC strips were developed from square Whatman paper, and the EC was made on baking paper treated with polydimethylsiloxane (PDMS). The PEDOT/PB-DMFCs operate in a passive mode with a few microliters of diluted methanol. The biosensor layer was obtained on the anode ink (a composite of EDOT, oxidized multiwalled carbon nanotubes, and carbon black with platinum and ruthenium) by electropolymerizing 3,4-ethylenedioxythiophene (EDOT), *in situ*, in the presence of L1CAM. Each PEDOT/PB-DMFC single cell generates a voltage in the range of 0.3–0.35 V depending on the cell, and a five-cell stack delivers a 1.5–1.6 V voltage range when fed with 0.5 M methanol. The fabricated PEDOT/PB-DMFC/biosensor was calibrated against L1CAM, showing linear responses from 1.0 × 10^–12^ to 1.0 × 10^–8^ M with a detection limit of 1.17 × 10^–13^ M (single cell mode). When the EC was connected to the PEDOT/PB-DMFC device, a color gradient was observed. Overall, this work opens horizons to the use of biosensors even in places with energy scarcity and offers an alternative to reducing the current energy demand.

## 1 Introduction

Portable and flexible disposable electrochemical biosensors are emerging for rapid and accurate quantification/monitoring of circulating biomarkers^1−3^ due to their advantageous features, including the possibility of real-time measurements.^4,5^ Electrochemical systems require a recognition element, a transducer, and an energy source,^6−8^ involving different (nano)materials and fabrication techniques,^9−11^ with flexibility being a key factor.^12,13^ Among various substrates that can be used to fabricate flexible and disposable biosensors, such as polyimide, PET, or PVC, paper seems to be the most promising as it is cheap, abundant, biodegradable, biocompatible, flexible, and can be easily modified.^14,15^ The combination of paper-based substrates, conductive nanoinks, and electrochemical detection has become popular with different setups, configurations, and detection modes.^16,17^ The nanoinks contain a conductive material, a binder, and a solvent.^18,19^ Conductive materials include mainly metallic nanoparticles (e.g., platinum, silver, copper)^20^ and/or carbon structures (e.g., carbon black, carbon nanotubes, graphene)^21,22^ or conductive polymers.^23^ However, the goal of portability is to develop a biosensor that is a stand-alone device that does not require a power supply or transducer equipment.

As far as power supply is concerned, great progress has been made in this direction with biofuel cells, which have an enzymatic electrode whose operation depends on the amount of substrate present in the sample.^24^ A similar perspective exists with microbial fuel cells.^25^ However, the fact that enzymes or biological cells are involved in the production means that the sensor system has a low reproducibility/stability and lifetime. Using synthetic sensing systems may avoid these drawbacks. This is the case of modified paper strips of paper-based direct methanol fuel cells (PB-DMFCs) built around a simple commercial Whatman paper.^26^ A molecularly imprinted polymer (MIP) was used as the biological recognition element (sarcosine), which has good selectivity and reproducibility and high stability compared to biological/biochemical counterparts. However, the power and voltage output levels were low, limiting its implementation as a fully portable biosensor system. This could be further improved with highly conductive materials such as PEDOT and MWCNT-COOH to achieve higher stability, current, voltage, and sensitivity to the target.

Considering the transducing element, the use of an electrochromic cell may eliminate the need for a device, as the color formed is linked to the energy generated in the cell.^27^ Thus, the use of a PB-DMFC/biosensor stack with sufficient potential to connect and trigger an electrochemical cell can give the hybrid biosensor a self-signaling function. This concept has been shown to be possible, but using a complex mechanical fuel cell structure in combination with an inorganic electrochromic system of tungstate and tungsten oxide nanoparticles.^28^

To our knowledge, the combination of intrinsic current/self-signaling properties in a methanol fuel cell paper strip system has never been described before. This is the first work to report on a hybrid paper-based methanol fuel cell system in a 5-stack configuration and in conjunction with an EC for visual display (PEDOT/PB-DMFC/biosensor-EC). The PEDOT/PB-DMFC/biosensor stack was connected to an organic electrochemical cell made of PEDOT:PSS, which was also developed on a single layer paper strip, requires no additional components, and is operated with a few microliters of an aqueous electrolyte. This PEDOT/PB-DMFC/biosensor-EC functions like a conventional passive DMFC, which is suitable for portable power generation, especially for small devices with low power requirements,^29−31^ such as the system presented here. The different properties of PEDOT as a conductive^32^ and electrochromic material^33^ have been investigated and optimized. This combination was developed here to monitor the interaction of the recombinant protein (L1CAM) with the PEDOT/PB-DMFC/biosensor stack by color change. The L1CAM protein is a cell adhesion molecule originally identified in the neuronal system and is currently associated with advanced stages of various cancers.^34−36^ Overexpression of L1CAM in advanced tumor stages and metastases has been reported,^37−39^ and this association with cancer progression makes L1CAM an important target. The setup was evaluated in terms of its analytical performance against L1CAM in buffer and Cormay serum solutions. The functionality of the system was tested in a single cell connected to a potentiostat and in a 5-stack array using the EC display (equipment-free).

## 2 Experimental Section

Detailed information on reagents, solutions, equipment, apparatus, and electrochemical and electrochromic assays is provided in the Supporting Information.

### 2.1 PEDOT Paper-Based Fuel Cell (PEDOT/PB-DMFC) Assembly

**Figure 1:**
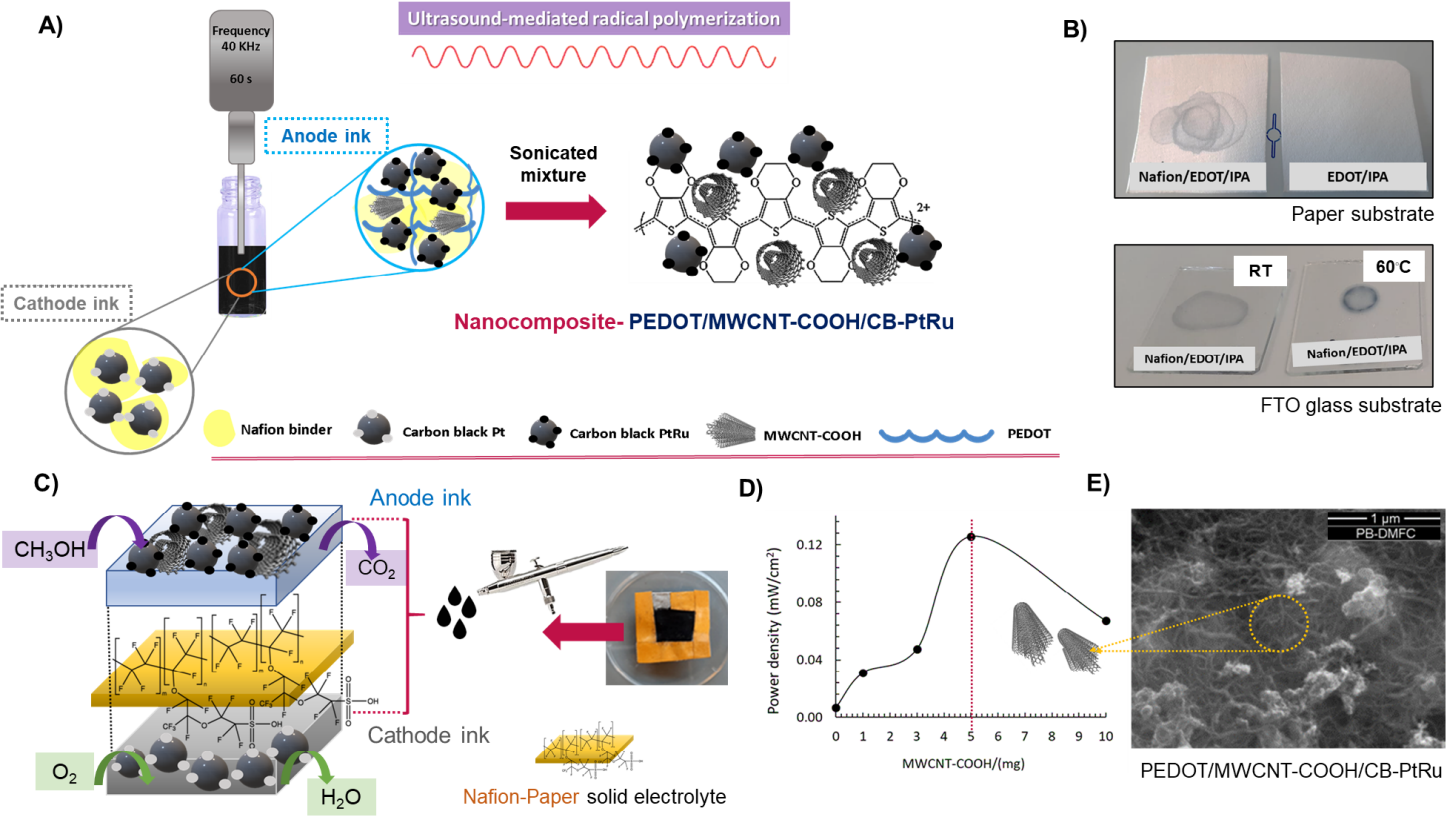
Schematic representation of the preparation of the anode/cathode inks for the PB-DMFC assembly using ultrasound with a microtip: the anode ink is composed of a mediated ultrasound synthesized nanocomposite of PEDOT/MWCNT-COOH/CB-PtRu dispersed in IPA in the presence of Nafion, and the cathode ink is obtained by ultrasonication of a solution of CB-Pt, Nafion, and IPA (A). Polymerization tests using two different substrates (Whatman paper and FTO glass) to drop-cast two ultrasonicated solutions: Nafion/EDOT/IPA and EDOT/IPA, evidencing the formation of a blue color in the sonicated solution containing Nafion compound, both at room temperature and at 60 ^◦^C (with temperature the coloration was more intense); in the absence of Nafion no coloration was observed in any substrate (at least in the 30 min of the experience duration) (B). General scheme of a typical PB-DMFC evidencing the Whatman paper impregnated with the Nafion electrolyte, which separates the anode and cathode, applied in both sides of the Whatman/Nafion electrolyte using the airbrush method (C). Maximum power density recorded using different PB-DMFCs assemblies in relation to the amount of MWCNTs (0–10 mg) in the ink formulation (D); top-view SEM image of PB-DMFC with PEDOT/MWCNT-COOH/CB-PtRu composite (E).

The paper-based PEDOT fuel cell platform (PEDOT/PB-DMFC) evolves from the prototype described in Carneiro et al.^26^ to boost significantly the output power of the fuel cell by combining it with conductive nanomaterials. This includes conductive polymers, carbon nanotubes (MWCNTs), or titanium(IV) oxide (TiO_2_) nanoparticles. In the first developed assembly,^26^ a layer of a paraffin solution diluted in IPA (10%) was applied to the anode electrode to hydrophobized the surface and reduce methanol crossover, which is not the ideal solution because it is an insulating compound that affects the final performance of the paper fuel cell platform. The development of this improved PEDOT/PB-DMFC involves several steps, including incorporation of the electrolyte, preparation, and deposition of the anode/cathode electrodes, impermeabilization of the surrounding paper region, and integration of the electrical connections (Figure S2). A general scheme for the fabrication of anode and cathode inks under an ultrasonic procedure is presented in Figure 1A. An improved anode nanoink, comprising a combination of EDOT and MWCNTs was developed by producing, *in situ*, a polymer nanocomposite based on PEDOT interaction with the ink components (CB-PtRu and Nafion) in a simple and fast way (60 s).

To the best of the authors’ knowledge, this is the first time that EDOT is polymerized in the described conditions, originating a PEDOT/MWCNT-COOH/CBPtRu nanocomposite. It is well-known that the formation of free radicals in an EDOT solution may initiate EDOT polymerization (depending on the conditions), and an ultrasonic treatment can induce the generation of radicals in certain materials, including the carbon materials and Nafion, herein used in the anode ink formulation. Ultrasonic treatment can promote the dispersion and functionalization of carbon nanomaterials,^40^ leading to the creation of defects and functional groups on their surface, which often result in the generation of radicals. These radicals, under the high frequency ultrasound can initiate the polymerization of EDOT monomer according the literature.^41−43^ In the case of Nafion, the ultrasonic waves lead to structural changes that also have the potential to generate radicals and trigger the polymerization of EDOT when exposed to the ultrasound treatment (Figure 1B).

Using two different substrates (Whatman paper and FTO glass), a few microliters of two EDOT solutions (0.01 M) were poured into drops: EDOT/IPA/Nafion and EDOT/IPA (in the absence of carbon materials and sonicated at the same time and frequency as the ink containing the carbon materials). When analyzing the results, the formation of blue color in the sonicated solution with the Nafion compound is observed for both substrates, both at room temperature and on a hot plate at 60 °C (the coloration becomes more intense with temperature). In the absence of Nafion in solution, no coloration was observed for any of the substrates (at least during the 30 min of the experimental period), so in addition to the initiation of EDOT polymerization by the free radicals in the carbon materials, a contribution from Nafion was also observed. The effect of high-power ultrasonic treatment of Nafion can produce chain end radicals with structures like R-O-CF2-CF2^●44^ and hydroxyl radicals (OH^●^)^45^ as already identified in literature. Also, PEDOT:PSS was stabilized by the sulfonic groups present in the PSS polymer through charge balance.^46^ The Nafion also contains sulfonic groups in its structure, which may increase the affinity of these two compounds in the solution.^47^ Therefore, this interaction alone is not beneficial for the best performance of the developed PEDOT/PB-DMFCs because the EDOT/Nafion interaction and/or Nafion degradation hinder the good functioning of the fuel cell system. The Nafion solution added to the inks has a dual function in fuel cell devices (conventional fuel cells or this particular paper fuel cell): it serves as a binder and also as a proton conductor.^46^ Modification of the Nafion by ultrasound or by interaction with EDOT affects the proton conductivity of the anode, resulting in low power fuel cells that are very difficult to activate (Table S1). To avoid this problem, the Nafion solution was added to the anode ink after sonication of all components and sonicated for only 10 s to prevent interaction with the unpolymerized EDOT. Thus, the final anode nanoink is formed by a synergetic composite mixture of CB-PtRu + MWCNT-COOH + EDOT + Nafion, prepared in IPA. The combination of these different materials allows obtaining the nanocomposite presented herein. The formation of these radicals depends on various factors, including the intensity and duration of ultrasonic treatment, the specific characteristics of the materials, and the surrounding conditions (e.g., solvent, temperature). These parameters were settled and controlled to minimize interferences and get a similar radical generation between the different experiments. For the cathode ink, a mixture of CB-Pt, Nafion, and IPA was sonicated, mixing all components simultaneously without affecting the fuel cell performance.

Both the anode and cathode inks were airbrushed onto Whatman paper modified with a Nafion electrolyte to separate the anode and cathode electrodes. A general scheme to better understand the experimental setup and the chemical reactions taking place is shown in Figure 1C. During preparation of the anode ink, gelation occurs due to the presence of EDOT in the ink; EDOT polymerizes under ultrasonic impact. Therefore, a volume of 300 μL of IPA was added before printing the anode ink to reduce the viscosity of the ink and prevent clogging of the airbrush. The cathode ink was painted on the other side of Whatman/Nafion directly from the ultrasonic device (without IPA dilution). Apart from the precautions taken in the preparation of the anode ink, an increase in hydrophobicity was observed in the anode electrode, limiting the spread of methanol on the surface of the electrode and thus the adsorption of methanol molecules on the Pt catalyst. To address this issue, the integration of ‘spacers’ into the ink formulation was investigated, testing TiO_2_ nanoparticles and MWCNT-COOH. The best results in terms of generated power of PEDOT/PB-DMFCs were obtained with the integration of 5 mg of MWCNT-COOH into the ink formulation, comparing the power density output in a range of (0–10 mg) integration of this nanomaterial into the anode inks (Figure 1D). Doping the ink with a higher amount of nanotubes (10 mg) leads to a reduction in electrochemical performance, which could be related to the fact that nanotubes are not catalytically active for methanol oxidation, and high amounts restrict the access of methanol to the Pt nanoparticles, which impairs methanol oxidation and consequently reduces the power of the fuel cell.

In a SEM image topography, the nanotubes are uniformly distributed on the surface of the anode electrode and act as ‘spacers’ to help limit the interaction between EDOT-Pt and Nafion and improve the conductivity of the system (Figure 1E). The results of these tests are summarized in Table 1 and discussed in Section 3.1.1.

**Table 1:** Summary of the Electrochemical Characterization of the Different PB-DMFCs Assemblies Produced in this Work and Comparison with Prototype 1

PB-DMFC	OCV (V)	power_Max_ (μW/cm^2^)	Ohmic resistance (Ω)	hydrophobic element	activation	stability (n◦ of pol. curves)
PB-DMFC-[26]	0.49	22.9	355	paraffin layer	fast	6–8
PB-DMFC/PTFE-1	0.46	23.8	900	PTFE layer (50 μL)	fast	6–8
PB-DMFC/PTFE-2	0.38	18.5	360	PTFE layer (25 μL)	fast	6–8
PB-DMFC/PEDOT-1	0.44	18.7	1300	PEDOT layer (chrono)	moderate	12–14
PB-DMFC/PEDOT-2	0.47	28.3	450	PEDOT layer (CV)	moderate	10–12
PB-DMFC/PEDOT-3	0.40	11.8	480	nanoink (CB-PtRu+ PEDOT + Nafion) + CV (PBS)	moderate	8–10
PB-DMFC/PEDOT-4	0.35	4.60	1550	*nanoink (CB-PtRu + EDOT 0.02 M + IPA + H_2_O)	slow	12–14
PB-DMFC/PEDOT-5	0.38	8.50	500	nanoink 4* – 2× diluted in IPA	slow	8–10
PB-DMFC/PEDOT-6	0.48	23.4	190	nanoink 4* – 3x diluted in IPA and Nafion added after sonication (10 s)	slow	8–10
PB-DMFC/PEDOT-7	0.42	16.5	570	PEDOT layer on anode surface (100 μL in H_2_O)	slow	10–12
PB-DMFC/PEDOT-8	0.28	12.4	710	PEDOT layer on anode and cathode surface (100 μL in H2O)	moderate	10–12
PB-DMFC/PEDOT-9	0.41	29.9	165	nanoink 4* – 2× diluted in IPA + 5 mg Tio_2_	moderate	4–6
PB-DMFC/PEDOT-10	0.38	12.0	420	nanoink 4* – 2× diluted in IPA + 10 mg Tio_2_	moderate	4–6
PB-DMFC/PEDOT-11	0.40	67.1	95	nanoink 4* – 2× diluted in IPA + 10 mg MWCNT-COOH	fast	2–4
PB-DMFC/PEDOT-12	0.41	125.7	75	nanoink 4* – 2× diluted in IPA + 5 mg MWCNT-COOH	fast	2–4
PB-DMFC/PEDOT-13	0.40	31.0	280	nanoink 4* – 2× diluted in IPA + 1 mg MWCNT-COOH	fast	4–6

### 2.2 PEDOT Biosensor Element Preparation

The biosensor for L1CAM detection was anchored to the top of the anode electrode of the PEDOT/PB-DMFC assembly using a 3-electrode system to perform electropolymerization of a solution of EDOT (0.01 M) in PBS buffer (previously rinsed with N_2_). The electropolymerization protocol should be performed immediately after the preparation of PEDOT/PB-DMFC to allow any free EDOT present on the anode surface to react with the polymerizing EDOT. This process results in a stable system where methanol crossover is controlled, eliminating the need of a paraffin layer.^48^ This developed biosensor was made of a PEDOT polymer, which comprises functions of a semiconductor, hydrophobic agent, and biorecognition element.

The first step of biosensor element preparation is the incubation of L1CAM (1.0 × 10^–7^ M) in the freshly prepared anode for *t* = 2 h. Then, the PEDOT/PB-DMFC was carefully washed with ultrapure water, dried with N_2_, and incorporated into the 3-electrode system. Electropolymerization was performed by applying 5–10 cycles of electrode potential sweep (50 mV/s) from −0.3 to 1.2 V and back to −0.3 V as a sweep rate of 50 mV/s. The same CV protocol was used to remove the L1CAM template, but instead of an EDOT solution, a 0.5 M H_2_SO_4_ solution was used, resulting in PEDOT/PB-DMFC/MIP with binding sites for L1CAM rebinding. In parallel, a nonimprinted PEDOT/PB-DMFC/NIP was developed using the same conditions and steps, except for the incubation of the L1CAM template. The polymer layer obtained by electropolymerization is porous and requires more time for the methanol to reach the Pt catalyst. Therefore, the polymer modified MIP and NIP samples needed more time for activation and stabilization. In addition, the PEDOT/PB-DMFCs modified with MIP and NIP were not reusable after calibration. The original current density was not retained, and the electrodes were discarded at the end of each calibration.

### 2.3 Electrochromic Cell (EC) Preparation

**Figure 2:**
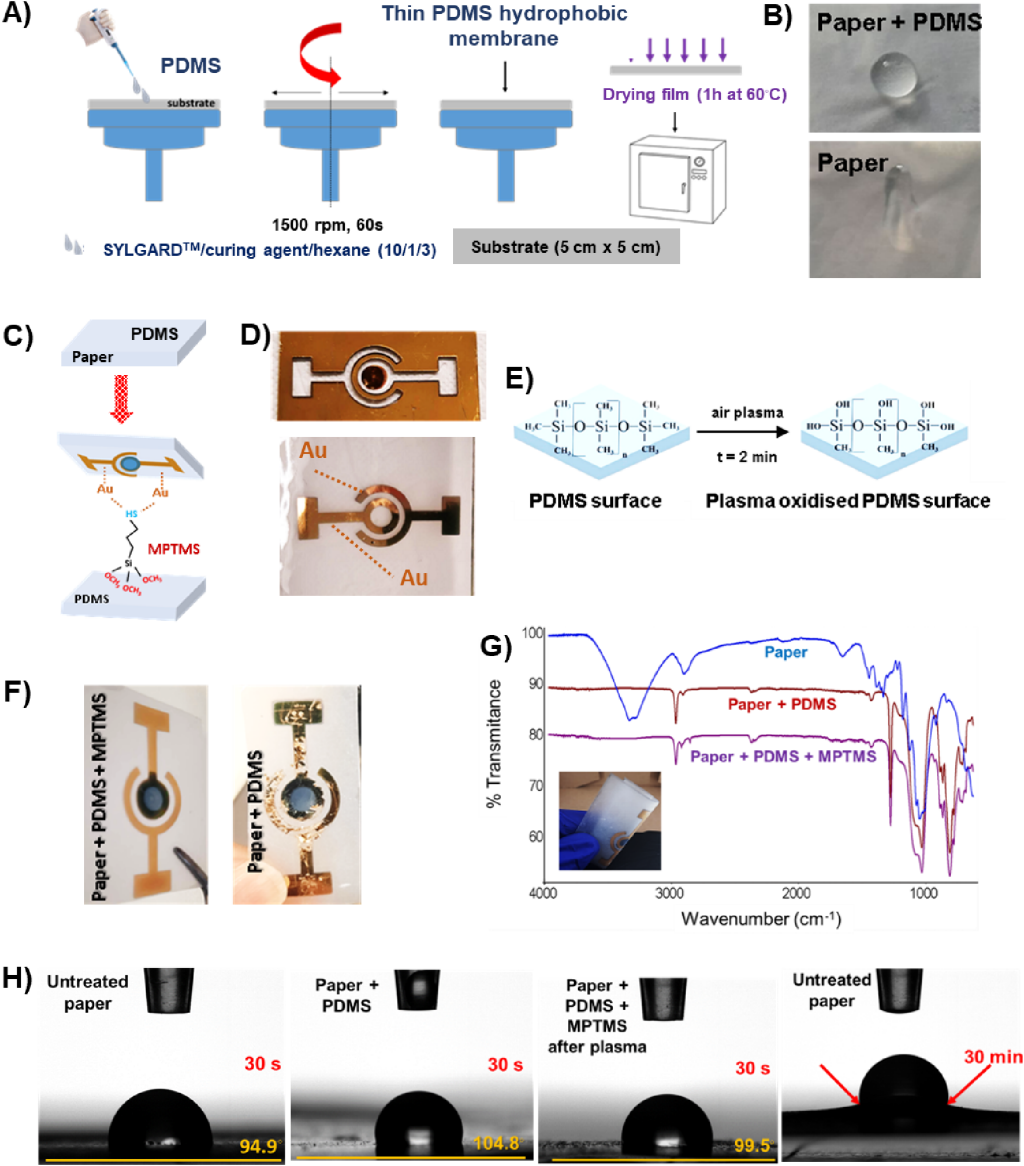
Schematic representation of the steps for EC device preparation and its characterization. The steps involved consisted of PDMS spreading in the paper surface by spin coating (1500 rpm, 60 s) and curing at 60 ^◦^C (A) to obtain a hydrophobic paper/PDMS compared to the untreated paper (B). Adhesion promotor (MPTMS) anchoring in the paper/PDMS to improve binding of gold and PEDOT/PSS (C); gold electrical connections obtained after sputtering using a glass mask (D); a plasma treatment (inclusion of hydroxide groups) was applied in the EC surface to decrease hydrophobicity of the support, essential to bind PEDOT/PSS electrochromic compound (E). Differences were observed in the presence and absence of MPTMS, after an electrochemical reading in terms of gold adhesion (F). FTIR–ATR analysis was used to follow the chemical modifications observed in the untreated paper, paper + PDMS, and paper + PDMS + MPTMS (G). Contact angle determination was also used to follow the water wettability and stability of the untreated paper and modified treated papers (H).

The paper substrate EC was developed on a translucid baking paper selected for its suitable properties, such as homogeneity, light transparency, and flexibility. The steps for the proper modification of the paper substrate are schematically shown in Figure 2.

Paper strips (5 cm × 5 cm) were PDMS covered by spin coating (1500 rpm, 60 s) of PDMS precursor solution and left in an oven at 60 ^◦^C for 1 h to cure the polymer (Figure 2A). The PDMS provides a hydrophobic layer (Figure 2B), which later supports the electrical connections and electrochromic material. After PDMS curing, the gold adhesion promoter (MPTMS) was spin-coated on the surface of the paper/PDMS (50 μL, spin coating: 800 rpm, 30 s), and the final paper/PDMS/MPTMS was dried at room temperature for 24 h (Figure 2C). Metallic contacts were deposited by sputtering a ca. 120 nm gold layer throughout a glass mask attached to the paper (Figure 2D). After gold deposition, plasma treatment (2 min) was performed (Figure 2E) to reduce the hydrophobicity of the surface, and a few microliters of a PEDOT:PSS dispersion diluted in dimethyl sulfoxide (DMSO, 95/5%)) was dispersed into the middle ring of the EC. Finally, the ECs were dried on a hot plate at 120 ^◦^C for 15 min and stored in a dark environment at room temperature before use.

## 3 Results and Discussion

### 3.1 PEDOT/PB-DMFC Device

#### 3.1.1 Set-Up Assembly Configuration and Characterization

**Figure 3:**
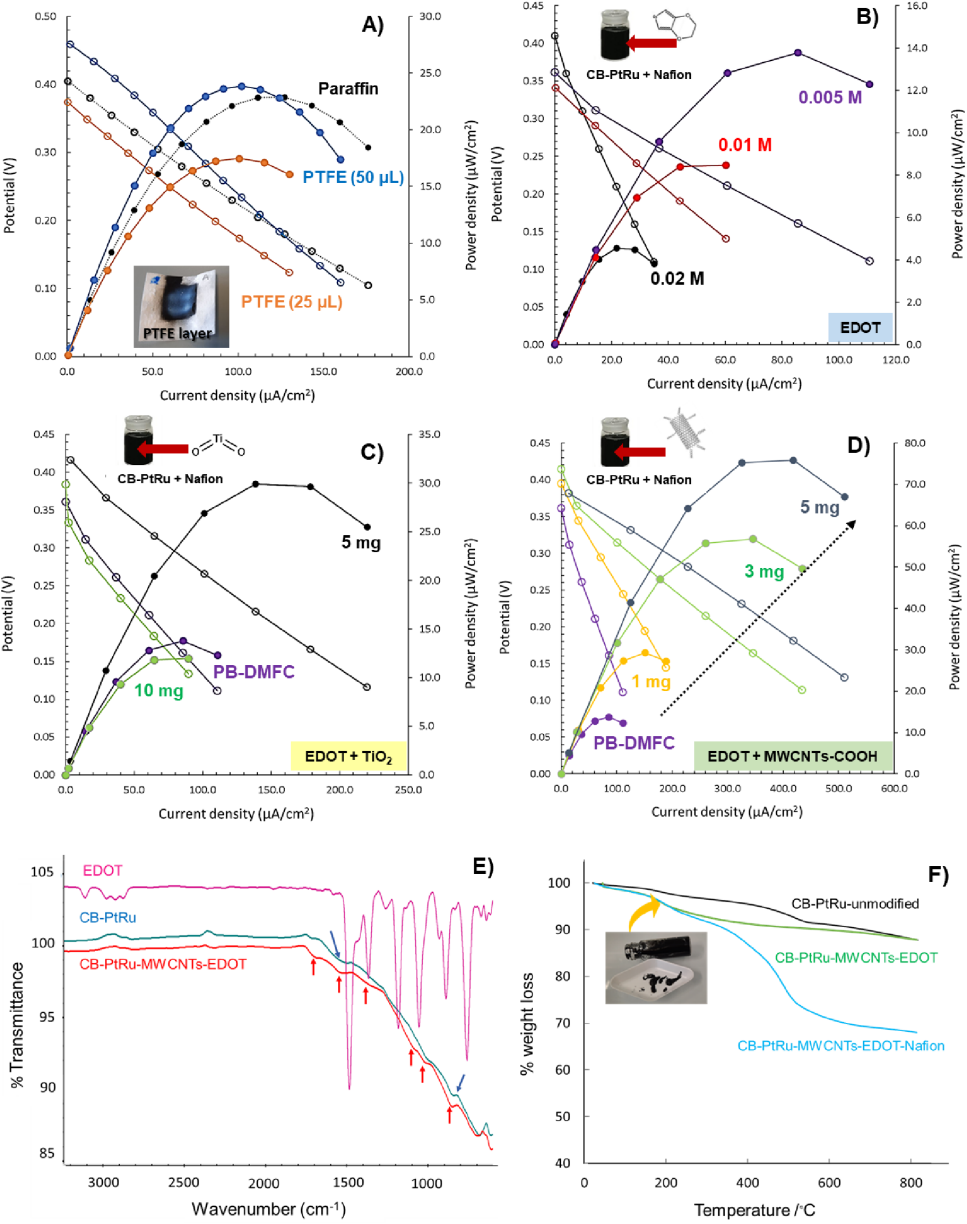
Electrochemical data of PB-DMFCs in terms of polarization (empty symbols) and power curves (full symbols) obtained with different nanoink formulations combining: hydrophobic components (layer of paraffin and PTFE) (A) and different concentrations of EDOT monomer in the ink formulations, before applying ultrasonication (B), doped with different amounts of TiO_2_ (C) and doped with different amounts of MWCNT-COOH (D). Characterization results by FTIR–ATR of the PEDOT–CB-PtRu powder nanocomposite obtained by ultrasonication in comparison with the EDOT monomer and commercial CB-PtRu powder (E). Thermogravimetric analysis of the different steps involved in the anode nanoink preparation: commercial CB-PtRu powder (unmodified), CB-PtRu–MWCNTs–EDOT ultrasonicated in IPA until ‘gelification’ of the sample (30 s), and CB-PtRu–MWCNTs–EDOT-Nafion upon ultrasonication during 15 s (F).

The PEDOT/PB-DMFC developed herein involves an innovative anode nanoink formulation, which was modified by a nanocomposite containing MWCNT-COOH and EDOT obtained *in situ* by ultrasonication. Figure S2 shows the different steps for preparing PB-DMFCs in this work. The focus of this part of the work is on the development of a suitable nanoink to modify the anode to obtain an assembly able to deliver a higher power density. The improvement in power density is related to the requirement of connecting the fuel cell stack to a signaling platform to get a fully portable, self-powered, and self-signaling paper-based device. The composition of the anode ink was selected as a target for improvement because it has the important function of hosting the biosensor element on its surface. Prototypes made from different formulations of the anode ink were tested to find the best combination that would provide PB-DMFC with higher electrical performance, stability, and ease of application by brush painting. The results are displayed in Table 1 and the polarization/power curves are shown in Figure 3A–D; an example of the calculations is shown in Figure S3 and tabulated in Table S1.

Various attempts have been made to improve the electrochemical parameters (OCV, maximum power/current density, and Ohmic resistance) by combining hydrophobic compounds (polytetrafluoroethylene, PTFE) and conductive polymers (PEDOT). When applying a layer of the hydrophobic agent PTFE, the electrochemical parameters are similar to those obtained when using paraffin (Figure 3A). These results were expected since PTFE contributes to surface hydrophobicity, but its insulating properties are negatively reflected in the final performance of the system. Although the use of PTFE in the regular PEM fuel cell inks seems to be promising,^48^ in this PB-DMFC system the combination with hydrophobic insulating compounds was not the best choice.

Several configurations with a conductive PEDOT polymer were tested. Conductive layers obtained by *in situ* electropolymerization or by preparing different PEDOT-based nanoinks were used. PEDOT was selected as a conductive polymer due to its compatibility with fuel cell electrodes, mild hydrophobicity, stability, and conductivity.^49−51^ The developed PB-DMFC/PEDOT-1,2 modified by conductive layers obtained by *in situ* electropolymerization did not show significant improvements in terms of the OCV, power output, and stability. As observed during the development of the biosensor layer, the PEDOT film on the anode electrode seems to block some platinum nanoparticles, meaning that methanol oxidation is delayed/hindered. When the EDOT monomer was added directly to the nanoink formulation, the opposite behavior was observed. The presence of EDOT in the ink formulation leads to PB-DMFCs with poor electrochemical performance and are very difficult to activate (Figure 3B). This result seems to be confusing when compared with the previously obtained ones. The analysis of all parameters and fuel cell behavior shows that the hydrophobicity of the EDOT monomer and the interaction with the sulfone groups of the Nafion binder affect the MeOH oxidation and proton conductivity, respectively. These interactions have also been confirmed in other studies.^52^ To compensate for these undesirable interactions, two dopants (TiO_2_, MWCNT-COOH) were selected and added to the nanoink formulations. The idea of these dopants is to act as spacers and prevent undesirable interactions that affect the electrochemical performance of the anode. With the combination of 5 mg TiO_2_, a considerable improvement in the electrochemical parameters is observed compared to the first PB-DMFC prototype, but a larger difference is observed compared to the ink without TiO_2_ dopant (Figure 3C). When 5 mg of MWCNT-COOH was added to the ink, a huge improvement in performance (Figure 3D) and faster start-up of the paper fuel cell was observed.

This final nanoink formulation was characterized by FTIR–ATR (Figure 3E) and TG (Figure 3F). Comparing the spectra of the CB-PtRu–MWCNTs–EDOT (red line) with those of the CB-PtRu sample (blue line), one can observe the appearance of low intensity bands in the nanocomposite spectra (Figure 3E), mainly due to the contribution of the EDOT molecule (pink line), since the carbon species have almost no characteristic vibrational bands (only carbon–carbon bonds). Analyzing the spectra of the CB-PtRu–MWCNTs–EDOT sample, one can observe the appearance of weak, intense bands at 848, 1050, 1150, 1480, and 1509 cm^–1^, which are characteristic of the vibrational spectra of the EDOT;^53^ a small band at 1700 cm^–1^ also is perceptible and can be derived from the −COOH of the oxidized MWCNTs.^54^

The TGA thermograms of CB-PtRu, CB-PtRu–MWCNTs–EDOT, and CB-PtRu–MWCNTs–EDOT–Nafion were also obtained to follow the different steps of the nanoink formulation. The results are shown in Figure 3F, evidencing the different thermal decomposition behaviors of the different analyzed samples. Analyzing the corresponding weight loss curves, it can be observed that the CB-PtRu–MWCNTs–EDOT–Nafion sample shows higher weight loss when compared to the unmodified CB-PtRu and CB-PtRu–MWCNTs–EDOT. This result is to be expected since in the other two samples investigated the major component is carbon, which has a low degradation profile in the range of temperatures evaluated. Thus, all samples can be considered thermally stable with a minimum percentage of weight loss occurring up to 200 ^◦^C.

These results support the preparation of a solid nanocomposite (PEDOT/MWCNT-COOH/CB-PtRu) formed by the ultrasound technique, which allows a perfect combination of the desired properties of each component. In this case, the inherent hydrophobicity of the PEDOT polymer^47^ replaces the insulating paraffin element and helps to control the methanol transfer from the anode to the cathode electrode and also increases the electrical conductivity of the anode surface. This discovery is important for the improvement of this simple paper fuel cell system, but it could also be significant for the application in regular DMFC systems since methanol crossover is still a current problem of this type of alcohol cell, which hinders its application and diffusion as an energy source.^55^ MWCNT-COOH added to the ink contributes as a spacer and radical donor and also increases the electrical conductivity. To achieve these better performance results, the PEDOT/MWCNT-COOH/CB-PtRu nanocomposite should be synthesized in a 3-step ink fabrication method: 1 – stabilization of CB-PtRu + MWCNT-COOH in IPA (24 h); 2 – addition of EDOT solution and sonication for 30 s to form a solid material; 3 – addition of Nafion and IPA (100 μL) and sonication for 10 s to form an inovative solid matrix. This last step avoids unwanted interactions of Nafion with EDOT, as Nafion is essential for ensuring the proton conduction of the fuel cell system. The final PEDOT/MWCNT-COOH/CB-PtRu nanocomposite is then diluted in an appropriate amount of IPA before brush application. The volume required to obtain the better properties of this material was also investigated and it was found that a higher dilution factor (5×) results in a low viscosity ink with medium performance and a lower dilution factor (1×) results in a high viscosity ink that is difficult to spread using the brush technique. To obtain a processable and well-tolerated nanoink for anode electrode fabrication, the nanocomposite must be diluted 3× in IPA.

#### 3.1.2 Sensing Layer Development and Characterization

**Figure 4:**
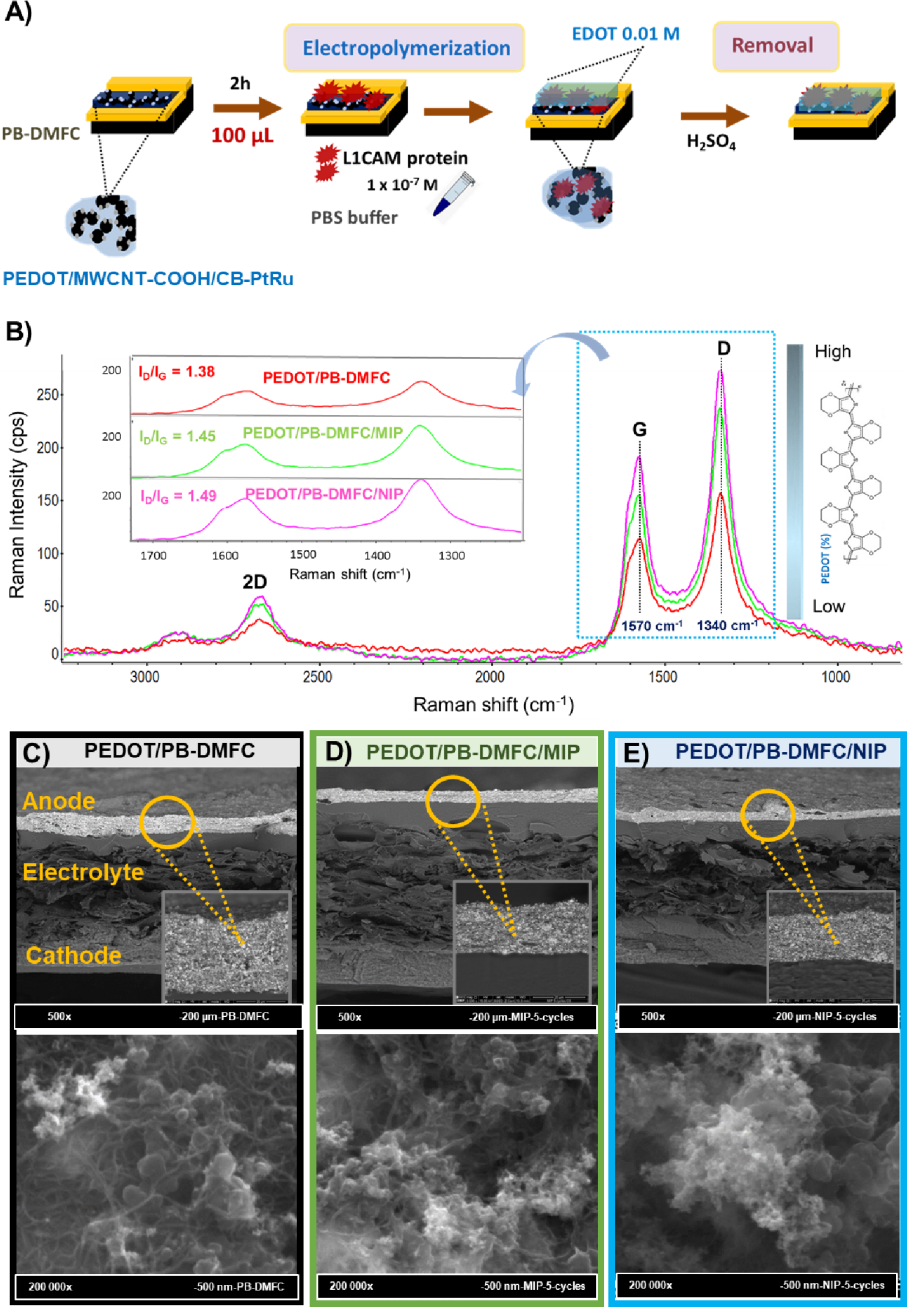
Steps involved in the development of a PEDOT/PB-DMFC/biosensor by electropolymerization, evidencing the interactions of the PEDOT-based anode with the EDOT solution in the MIP formulation (A) and characterization results obtained by Raman analysis at 532 nm of the different PEDOT/PB-DMFC assemblies (PEDOT/PB-DMFC/MIP and PEDOT/PB-DMFC/NIP) with the corresponding *I*_D_/*I*_G_ calculated ratios (inset) (B); SEM images in cross section and topography of a PEDOT/PB-DMFC (C), and electropolymerized samples to obtain the biosensor function (PEDOT/PB-DMFC/MIP) (D) and the control (PEDOT/PB-DMFC/NIP) (E).

The sensing layer was built up on the surface of the PEDOT/MWCNT-COOH/CB-PtRu anode immediately after the electrode was painted (Figure 4A). The idea is that the free EDOT monomer present on the anode surface can react with the EDOT solution monomer in the electropolymerization protocol, resulting in the PEDOT/PB-DMFC/biosensor. This process makes it possible to obtain a hybrid nanocomposite carbon electrode with dual functions: methanol oxidation for energy production and sensing properties. The working principle of this hybrid system is related to the ability of methanol molecules to reach the catalytic sites of platinum and be oxidized by them. In a PEDOT/PB-DMFC, the active sites of the Pt are fully available to react with the methanol molecules, while in the PEDOT/PB-DMFC/biosensor, the sites of the Pt catalyst are less accessible due to the fact that Pt nanoparticles are covered with porous polymer film. The porosity in the polymer film is formed during MIP production (see Section 2.2); the voids in the polymer are geometrically designed at the molecular level to have a complementary shape to the target sensor substance, L1CAM. The voids present in the polymer film allow the methanol to spread on the Pt catalyst surface, but when the protein rebounds in the MIP formed voids, the methanol molecules are prevented from reaching the catalyst; less methanol oxidation takes place. This phenomenon leads to a decrease in the potential and performance of the system, which is correlated with the biosensor response.^56^

The different developed PEDOT/PB-DMFCs were analyzed by Raman spectroscopy to follow the surface changes of the different anode electrodes (Figure 4B). The spectra obtained show differences in the intensity of bands G (1570 cm^–1^) and D (1340 cm^–1^), which are more pronounced for the PEDOT/PB-DMFC/NIP control, the anode electrode with a higher PEDOT loading. For the PEDOT/PB-DMFC/MIP, the anchoring of the electropolymerized PEDOT is hindered by the presence of the L1CAM previously incubated on the anode surface. These results were also confirmed by analyzing the cyclic voltammograms of PEDOT/PB-DMFC/NIP and PEDOT/PB-DMFC/MIP obtained during the electropolymerization protocol (Figure S4). The relative intensity of the D and G bands can be an indicator of surface modifications in a given carbon material and reveal the degree of disorder in a sample. The *I*_D_/*I*_G_ was calculated for both fuel cells and an *I*_D_/*I*_G_ ratio of 1.38 was obtained for the PEDOT/PB-DMFC. This value is significantly higher when compared to a PB-DMFC without EDOT in the ink formulation (0.91), which is further evidence that this developed nanoink is significantly different from the ink applied in the first prototype.^26^ The *I*_D_/*I*_G_ calculated for PEDOT/PB-DMFC/MIP is 1.45, while it is 1.49 for PEDOT/PB-DMFC/NIP. These results show that a higher percentage of PEDOT polymer on the surface leads to a more uniform and homogeneous surface, so the presence of the polymer anchored in the surface also prevents the interference of the PtRu metals present in the anode in the Raman spectra. Since the PtRu metals interfere with the intensity of the recorded bands, the sample with a higher PEDOT loading shows a higher intensity in both the G, D, and 2D bands. This difference in the intensity of the ratios is significant and originated form a uniform polymer layer anchored to the anode surface of both PEDOT/PB-DMFC arrays.

To evaluate these surface modifications, an SEM analysis is performed to assess the topography of the surface of the different anode electrodes as well as a cross-sectional analysis evaluation of the different PEDOT/PB-DMFCs. The analysis of the cross section obtained by SEM for all the fabricated experimental setups shows a uniform and well-defined 3-layer (anode–electrolyte–cathode) distribution for all the fabricated fuel cells. Interestingly, a decrease in layer thickness is observed for the PEDOT/PB-DMFC/MIP and PEDOT/PB-DMFC/NIP samples, indicating a more homogeneous surface. In the case of PEDOT/PB-DMFC, the presence of a network of MWCNT-COOH is readily apparent. In the analysis of the electropolymerized samples for the sensor (PEDOT/PB-DMFC/MIP) and the control (PEDOT/PB-DMFC/NIP), a visible surface change is observed, with less distinction between the different network components, especially in the case of PEDOT/PB-DMFC/NIP. To complete the surface characterization, a SEM analysis was also performed to follow the surface changes of PEDOT/PB-DMFC/MIP and PEDOT/PB-DMFC/NIP after electrochemical stabilization (Figure S5). The obtained results show a change in the roughness in both systems.

#### 3.1.3 Calibrations of the PEDOT/PB-DMFC/Biosensor (Single-Cell Configuration)

The PEDOT/PB-DMFC/biosensor and PEDOT/PB-DMFC/NIP assemblies were first activated by running multiple polarization curves until the systems reached maximum OCV and power density (the number of polarization curves required for full activation depends on the PEDOT/PB-DMFC setup and can range from 3 to 5 consecutive trials). In the single-cell configuration, calibrations were performed with the potentiostat. Once a polarization curve was established, the PEDOT/PB-DMFC/biosensor was washed with water, dried with N_2_ and 100 μL of a 0.5 M methanol solution was added to the anode layer. This procedure was repeated continuously throughout the calibration experiment. A prior stabilization in the background medium used was performed to ensure that the measured electrical signal was due to the L1CAM interaction and not to the medium in which the standard solutions were prepared. Two background media were used to prepare the L1CAM standards: MES Buffer and pretreated human serum (Cormay). In the case of human serum (Cormay), a 100-fold dilution in a buffer was performed to achieve signal stability. This stability procedure was performed by incubating the sensory layer successively with the same volume of background medium used for the incubation of the L1CAM standards (100 μL) until a stable electrical signal was achieved (deviations of less than 2%). Figure S6 shows an example of a stabilization protocol with MES buffer and diluted Cormay serum. It shows that the successive MES incubations hardly affect the signal, unlike the Cormay serum, which needs to be pretreated before use in the sensor area. It is therefore important to emphasize that the pretreatment of the Cormay serum serves to remove excess salts and to preserve the proteins. This pretreatment of a real sample does not limit the use of this system as a portable POC instrument, as it is a simple procedure to remove the salts and dilute the sample. For example, the COVID test strips developed by homemade analyses also require dilution in a buffer medium before use in the sensor area. Therefore, selectivity tests were also performed to evaluate the performance of this PEDOT/PB-DMFC/biosensor in the presence of interfering proteins.

**Figure 5:**
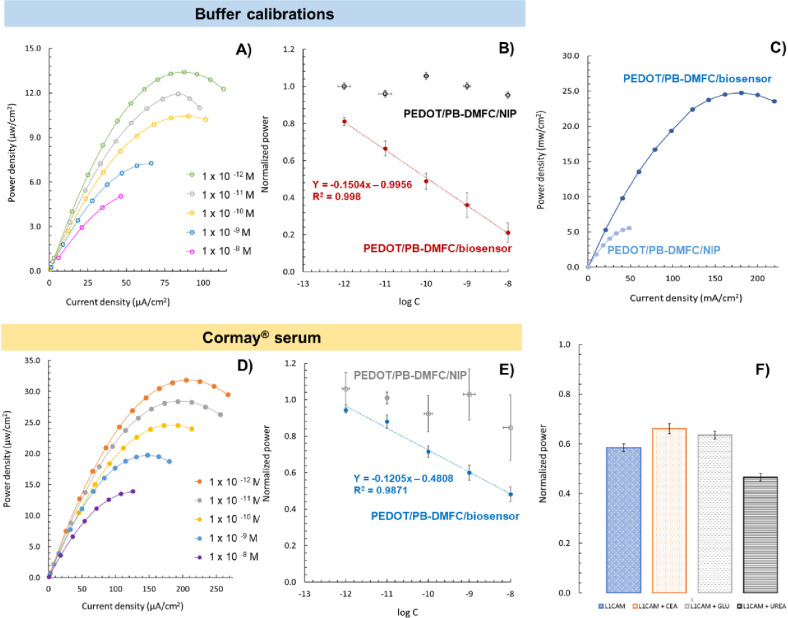
Power–current density curves obtained during a calibration after incubation of L1CAM standards of increasing concentrations prepared in buffer (A), with the respective calibration curve compared with a control experiment (PEDOT/PB-DMFC/NIP) in the range 1.0 × 10^–12^–1.0 × 10^–8^ M (B); comparison of the recorded power–current density curves of a pair of PEDOT/PB-DMFC/biosensor and PEDOT/PB-DMFC/NIP obtained in the same batch, showing the effect of the polymer cavities in the power produced by the two different setups (C). Power–current density curves were obtained during a calibration with L1CAM standard solutions prepared in cormay serum in the same concentration range used in buffer (D), with the respective calibration curve compared with the control PEDOT/PB-DMFC/NIP sample (E). All calibration curves were drawn using the average of the maximum power density obtained at each L1CAM concentration. Selectivity assay results (buffer) obtained using independent PEDOT/PB-DMFC/biosensor strips after incubation of a L1CAM standard solution (1.0 × 10^–10^ M) and L1CAM spiked with interfering compounds [carcinoembryonic antigen (CEA), glucose (GLU), and urea] with the respective interference analysis presented in normalized values (F).

After stabilizing the signal from the PEDOT/PB-DMFC/biosensor and, in parallel, the control PEDOT/PB-DMFC/NIP in appropriate media, successively increasing concentrations of L1CAM standard solutions in the range of 1.0 × 10^–12^–1.0 × 10^–8^ M were incubated directly on the surface of the anode sensor layer; the polarization curve was followed after incubation of each concentration (from the OCV potential to a predefined stop potential of 0.1 V). It is important to note that the initial OCV of the polarization curve decreases as the calibration experiment progresses, while the sensor loses the ability to return to initial values when the L1CAM is trapped in the polymer voids. However, the time required to reach a stable OCV varies during the calibration but must be determined at the beginning of the experiment and maintained until the end of the calibration, as the kinetics of methanol oxidation are time-dependent and a long contact time favors that a small molecule such as methanol can easily enter the polymer and influence the results. This does not affect the sensor analysis and response, especially in this developed PEDOT/PB-DMFC/biosensor, as the pregnancy tests and COVID tests were also time dependent (the result is valid for only a short and specific period of time). Each L1CAM standard solution was left on the anode surface for 20 min and covered with a square glass to ensure efficient distribution of the sample over the entire sensory surface and also to prevent evaporation. After this time, a 0.5 M MeOH solution was spread on the anode side, followed by the electrochemical measurement (OCV stabilization + sampling DC voltammetry technique). This procedure was performed in parallel with a control fuel cell (PEDOT/PB-DMFC/NIP) to monitor the nonspecific interactions of L1CAM with the nonimprinted polymer. Several calibrations were performed to evaluate the analytical response of this hybrid paper biosensor strip. Figure 5A shows a typical plot of the performance curves obtained during a calibration procedure in buffer media of a PEDOT/PB-DMFC/biosensor, and Figure 5B shows the average calibration curves compared to those of the control (PEDOT/PB-DMFC/NIP). Figure 5C shows the differences in performance values recorded for a pair of PEDOT/PB-DMFC/biosensor and PEDOT/PB-DMFC/NIP from the same batch. This illustrates the differences between a paper fuel cell with a sensory anode integrating a polymer with cavities (PEDOT/PB-DMFC/biosensor) and a polymer without cavities in the polymer (PEDOT/PB-DMFC/NIP). The power generated is 5 times higher with the PEDOT/PB-DMFC/biosensor, as the cavities enable more efficient methanol enrichment and oxidation in the Pt catalysts.

Figure 5D shows a typical calibration graph obtained in pretreated Cormay serum with the corresponding average calibration curves in Figure 5E. These results are tabulated in Table S2.

Calibration data show that the average power density decreases linearly with successive concentrations of the L1CAM standards for the two media studied. Therefore, PEDOT/PB-DMFC can be tuned into a PEDOT/PB-DMFC/biosensor with activity dependent on the L1CAM concentration. Analysis of the calibrations performed in the MES buffer (Figure 5B) shows that the PEDOT/PB-DMFC/biosensor performance decreases by ∼70% compared to the maximum L1CAM concentration, compared to the initial value after the blank experiments (background stabilization), with a squared correlation coefficient of ∼0.998. This variation was generally consistent between the different calibrations performed in buffers using PEDOT/PB-DMFCs with similar initial power values. This result, compared to the first PB-DMFC prototype developed for sarcosine targeting,^26^ proves that this improved system integrating EDOT into the ink allows a higher impact in terms of power density dependence on target concentration, which traduces in a higher sensitivity of the system.

The limit of detection of this PEDOT/PB-DMFC/biosensor was 1.17 × 10^–13^ M and was calculated by applying the equation LoD = (*y*_blank_–3SD_blank_)/Slope. Comparing the results of the PEDOT/PB-DMFC/biosensor with the nonimprinted PEDOT/PB-DMFC/NIP, a random response is observed, indicating minor nonspecific interactions of L1CAM molecules. These correspond to the electrostatic interactions between the polymer film and the L1CAM, which are less favored than in the presence of binding sites (MIPs, where these are more intense and effective due to the spatial arrangement).

Analysis of calibrations performed in pretreated Cormay serum shows that the final PEDOT/PB-DMFC/biosensor signal output decreases by ∼50% (compared to the initial value after stabilization of the background medium) toward the maximum concentration of L1CAM. These results demonstrate the good performance of this paper-based biosensor, which also shows good sensitivity in more complex media such as Cormay serum. Therefore, the sensitivity in this complex matrix medium is not as high as that in buffer media, which is also a price to pay in more sensitive systems. The L1CAM target is thus a more complex molecule than sarcosine, so this result can be considered very promising, as the performance of the PEDOT/PB-DMFC/biosensor strip retains a higher impact on the potential, current density, and performance of the fuel cell strip.

The analysis of the nonspecific interactions in the control fuel cell (PEDOT/PB-DMFC/NIP) shows that the behavior of this system is random and the interactions are negligible in this medium. This polymer film of this control fuel cell was made in the absence of L1CAM, thus forming a polymer network without binding sites that do not selectively interact with L1CAM proteins. Moreover, this polymer layer also restricts methanol diffusion to the Pt catalyst nanoparticles, as it has no binding sites and is therefore less porous. This lower porosity limits the performance of the control fuel cell and makes it insensitive to sample incubation.

Thus, this self-supplied paper strip PEDOT/PB-DMFC/biosensor can be used for screening and monitoring the L1CAM biomarker, with the possibility of connecting it to an EC cell for outdoor use without being limited to standard analytical laboratory procedures. A cutoff value of 5.4 ng/mL for soluble L1CAM has been reported in the literature to define clinical risk groups associated with tumor progression.^57^ This concentration (2.6 × 10^–11^ M) can be detected on paper strips using PEDOT/PB-DMFC/biosensor, suggesting that this autonomous paper platform may be a useful tool for detecting abnormal L1CAM levels associated with tumor diagnosis. These data are related to sensors calibrated in the 24 h after their production. Tests made with different sensors stored in the dark until a maximum of 5 days maintained the analytical performance observed in the calibrations.

Selectivity tests of the PEDOT/PB-DMFC/biosensor were performed in buffer solutions against 3 major interfering species (carcinoembryonic antigen-CEA, glucose, and urea). The assays were performed with different PEDOT/PB-DMFC units, incubating a standard L1CAM solution (1.0 × 10^–10^ M) in a PEDOT/PB-DMFC/biosensor area. In parallel, the same procedure was repeated with different PEDOT/PB-DMFC/biosensor units by incubating the same concentration of L1CAM in the presence of the respective interfering substance. Both PEDOT/PB-DMFC/biosensors were analyzed using their respective polarization curves. The corresponding interference analysis is obtained from the normalized peak power between the average L1CAM response/average L1CAM + interference response. These data are shown in Figure 5F. They show that the signal deviation is low in the presence of the interfering species CEA and glucose, with the urea species causing the largest deviation from the L1CAM signal (20%). Therefore, these data confirm the high preference of this sensor for the L1CAM target molecule with signal changes of 8–20%, in both the positive and negative directions.

### 3.2 EC Assembly and Characterization

The electrochromic device was mounted in a single-layer semitransparent paper strip previously modified with PDMS (hydrophobic treatment and reinforcement) and MPTMS (adhesion promoter for Au and PEDOT:PSS). In the absence of the adhesion promoter MPTMS, the gold layer is not stable in the presence of the electrolyte and leaves the paper during the measurements. Figure 2F shows a real image after prolonged use of the ECs in the absence/presence of MPTMS and illustrates the differences in the adhesion of the sputtered gold film. The chemical characterization of the EC paper support and the final assembly was conducted using the FTIR-ATR (Figure 2G). In the unmodified paper strip sample, the characteristic absorption bands of cellulose are visible at 3330 cm^–1^ (stretching of hydroxyl groups), at 2906 and 1373 cm^–1^ (stretching and deformation vibrations of C–H) and the strong, intense band at 1027 cm^–1^ (stretching of −C–O group).^58^ When analyzing the paper modified with the PDMS layer, all the characteristic absorption bands of the PDMS molecule can be observed: 1257, 1008, and 786 cm^–1^, representing the functional groups of Si–CH_3_, O–Si–O, and Si–(CH_3_)_2_, respectively.^59^ The paper modified with PDMS and MPTMS shows two small bands around 2917 and 2849 cm^–1^, which can be assigned to the C–H stretching vibration of −CH_3_ of the MPTMS molecule.^60^ These results confirm that the paper was successfully modified for EC. To evaluate the waterproof properties of the EC paper strip, the dynamic contact angles were determined, and the results are shown in Figure 2H. When analyzing the results for untreated paper, paper + PDMS and paper + PDMS + MPTMS (after plasma treatment), differences in the water contact angle for the different paper strips can be observed. For untreated paper, the contact angle of the droplet in the first 30 s is 94.9^◦^. After 30 min, the drop spreads to the inside of the paper and damages the paper surface. The sample of paper + PDMS shows higher hydrophobicity with an increase of the contact angle to 104.8^◦^, which decreases to 99.5^◦^ after the plasma treatment protocol. These results show that the PDMS treatment increases the hydrophobicity of the paper strip, which helps to obtain a durable, resistant, and reusable EC paper strip.

**Figure 6:**
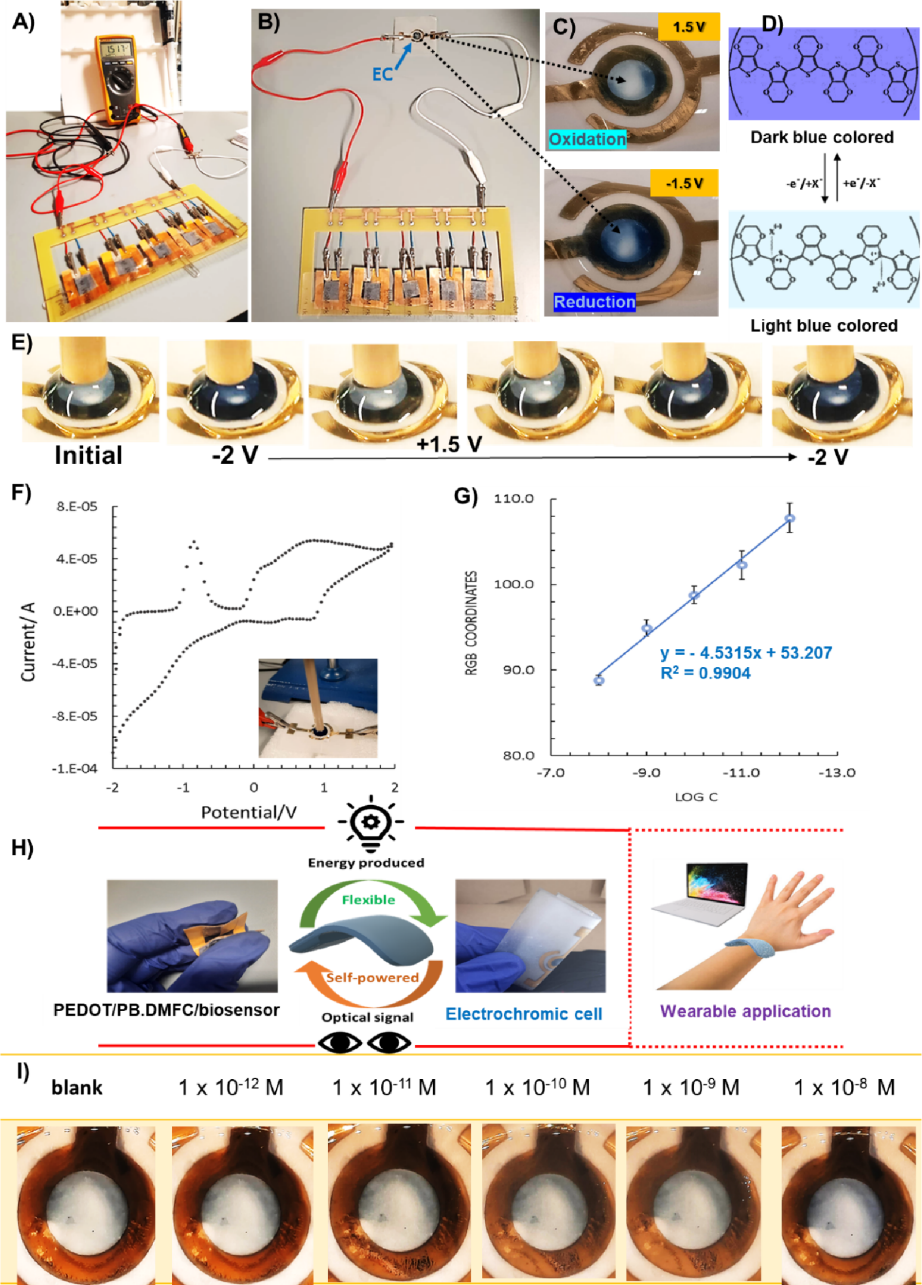
PEDOT/PB-DMFC/biosensor assembled in a five-stack configuration to produce a voltage of ∼1.5 V (recorded with a multimeter) (A), connected to the EC cell (B) to promote the color change of PEDOT:PSS polymer (C) from light to dark blue (inversion of polarization) (D). Color variation of PEDOT:PSS polymer obtained in three electrodes system (E) and the respective voltammogram in the range of −2 V to +2 V (F). Calibration of PEDOT/PB-DMFC/biosensor-EC showing the RGB color coordinates extracted from image J software (using blue coordinates) in three different spots (G). Schematic representation of the PEDOT/PB-DMFC/biosensor and EC evidencing the flexibility of each component and the overall proprieties suitable for wearable devices (H). Example of the pictures recorded during a calibration protocol after L1CAM incubation in the range of 1.0 × 10^–12^–1.0 × 10^–8^ M (I).

After the paper treatment, a layer of the electrochromic PEDOT:PSS polymer was built up; the electrochemical characterization was carried out in a 3-electrode system with LiClO_4_ as electrolyte. A color change from dark blue (−2 V) to light blue was observed, with a color gradient between the tested potentials (−2 to 2 V) (Figure 6E) with a reversible behavior. An example of an obtained voltammogram corresponding to one cycle is shown in Figure 6F. This single layer EC was suitable for integration with PEDOT/PB-DMFC/biosensor, resulting in a fully flexible, self-powered, and self-signaling paper-based sensor with potential application as a wearable biosensor (Figure 6H).

### 3.3 Assembly of the Portable System (PEDOT/PB-DMFC/Biosensor Stack) Interfaced with the EC

The PEDOT/PB-DMFC/biosensor-EC combines a stack with the ability to hold up to 5 fuel cell paper strips modified with the sensor function (Figure S7) and a PEDOT-based electrochromic cell. This portable prototype functions as a unique and fully autonomous sensing device and is lightweight, disposable, and potentially inexpensive to commercialize. The conductivity and electrochromic properties of PEDOT were explored to develop a simple, flexible, and device-free paper-based fuel cell biosensor (Figure 6A,B). Since the films prepared from the commercial PEDOT:PSS dispersion shows a color change from light to dark blue (Figure 6C), with different shades of blue observed over a wide range of potentials (−1.5 V, + 1.5 V), the PEDOT/PB-DMFC should generate enough potential to trigger the electrochromic device. The color change of PEDOT:PSS is related to two different states: an oxidized state, which is light blue, and a reduced state, which is dark blue (Figure 6D).^61^ With the developed PEDOT/PB-DMFC stack, it is possible to promote this color change by simply connecting the EC to the PEDOT/PB-DMFC stack.

In order to achieve a voltage of at least 1.5 V, a stack of fuel cell strips had to be used, as each one produces ca. 0.3 V. The stack was assembled and connected to the EC. Then methanol solution was added to the anode layers and left for 30 min until a stable potential (∼1.5 V) was reached, which was measured with a multimeter (Figure 6A). After this stabilization, the multimeter was replaced to the EC and a few microliters of lithium perchlorate were added to the EC. After 2 min, a photo was taken to record the initial state of the system; then, each PEDOT/PB-DMFC/biosensor was washed, dried with nitrogen and incubated with the buffer (the incubation of the buffer and standards was set to 20 min). After washing and adding the methanol fuel again to the PEDOT/PB-DMFC/biosensor anodes, a new photo was taken after 2 min. This time is more than sufficient to observe the complete color change (normally the EC, which is connected to the stack, changes color in less than 1 min). This procedure was repeated throughout the calibration and the standard concentrations tested were the same as those used in the single cell setup. The captured images were analyzed using ImageJ software. The quantitative data obtained from the color calibration are shown in Figure 6G. An example of the images taken during calibration is shown in Figure 6I. The color coordinates were determined at three different locations and the blue coordinate values were selected. When the blue RGB coordinates are plotted against the logarithm of the L1CAM concentration, a linear curve with a squared correlation coefficient of *R*^2^ = 0.9904 is obtained (original RGB results are shown in Table S3). Although the color change is subtle and not ideally visible to the naked eye (the blue tone became slightly darker due to the calibration), the program is sensitive to the changes, and it is possible to observe the linear behavior of the system with L1CAM detection by the developed sensor. In a future improvement, a dopant can be used in the PEDOT:PSS formulation to shift the dark blue color observed at negative potentials to the positive potentials where the color change is less pronounced.

## 4 Conclusions

The developed PEDOT/PB-DMFC/biosensor-EC device is an innovative and promising self-sufficient platform for clinically relevant L1CAM detection; it exploits methanol fuel cells as an energy source, a MIP sensing element, and an electrochromic indicator. The developed device is self-powered and self-signaling with all components fully assembled on paper substrates for the first time. The PEDOT/PB-DMFC/biosensor shows a linear sensing response within a diagnostically significant L1CAM concentration of 1.17 × 10^–13^ M. High sensing selectivity of the developed system was proved in the assays in Cormay serum.

Compared to AA batteries, it can be said that the current work has advantages and disadvantages. A fuel cell works until the fuel is available; it does not need to be recharged, and it is not harmful to the environment in terms of the products produced. This is the main advantage of this fuel cell sensor system, as it works with atmospheric oxygen and a very small amount of methanol. Thanks to this feature, it can be used anywhere, as it does not require an electrical energy source. Its disadvantage is the use of metal catalyst Pt, which is expensive and limited. However, Pt can be replaced by other metallic/organic catalysts, which are cheaper and more available.

Overall, the presented PEDOT/PB-DMFC/biosensor-EC prototype can be considered a promising and suitable tool for portable point-of-care applications and wearable biosensors with the ability to analyze L1CAM in a wide range of concentrations. This developed PEDOT/PB-DMFC/biosensor-EC can be classified as an innovative strategy in the development of device free and portable electrochemical biosensors, combining three emerging and powerful technologies (fuel cells, MIPs and electrochromic materials). Further improvements to the current platform (catalysts, fabrication processes, and fuels) could enable a low-cost and publicly available paper-based fuel cell biosensor for health monitoring and diagnostic applications.

## Supporting Information Available

Experimental section (additional information about reagents and solutions, equipment and apparatus, and electrochemical and electrochromic assays); additional figures (S1 to S7); and additional Tables (S1 to S3) (PDF)
