# Supplementary material for: Portable and Autonomous PEDOT-Modified Flexible Paper-Based Methanol Fuel Cell Sensing Platform Applied to L1CAM Recombinant Protein Detection
Source: ACS Appl Polym Mater. 2024 Mar 11;6(6):3207–21. doi: 10.1021/acsapm.3c02781 (PMC10964318; doi:10.1021/acsapm.3c02781)
Supplement: Supplementary file 1 — ap3c02781_si_001.pdf [file ap3c02781_si_001.pdf]

Supporting information

to

Portable and autonomous PEDOT modified flexible paper-based methanol fuel cell sensing platform applied to L1CAM recombinant protein detection

Liliana P. T. Carneiro[1,2,3], *Alexandra M. F. R. Pinto*[3,5], *Dzmitry Ivanou*[4,5], *Adélio M. Mendes*[4], *M. Goreti F. Sales**[1,2]

[1]BioMark, sensor research@UC/CEB, Department of Chemical Engineering, Faculty of Sciences and Technology, University of Coimbra, Rua Sílvio Lima, Pólo II, Coimbra 3030-790, Portugal

[2]BioMark, sensor research@ISEP/CEB, School of Engineering, Polytechnic Institute of Porto, Rua Dr. António Bernardino de Almeida, 431 Porto 4249-015, Portugal

[3]CEFT, Transport Phenomena Research Center, Department of Chemical Engineering, Faculty of Engineering, University of Porto, Rua Dr. Roberto Frias, Porto 4200-465, Portugal

[4]LEPABE - Laboratory for Process Engineering, Environment, Biotechnology and Energy, Faculty of Engineering, University of Porto, Rua Dr. Roberto Frias, Porto 4200-465, Portugal

[5]ALiCE - Associate Laboratory in Chemical Engineering, Faculty of Engineering, University of Porto, Rua Dr. Roberto Frias, Porto 4200-465, Portugal

* To whom the correspondence should be addressed. BioMark, Sensor Research/UC, Instituto Department of Chemical Engineering, Faculty of Sciences and Technology of Coimbra University, Rua Sílvio Lima, Polo II, 3030-790 Coimbra, Portugal. Tel.: +351239798733; E-mail: goreti.sales@gmail.com; goreti.sales@eq.uc.pt

TABLE OF CONTENTS

1. Experimental section

1.1. Reagents and solutions

1.2. Equipment and apparatus

1.3. Electrochemical and electrochromic assays

2. Supplementary Figures

- **Figure S1.** Pre-treatment steps of the human serum (Cormay®) for L1CAM standards preparation: (1) – add AgNO3 to the pure Cormay® serum; (2)- stir in a stirring plate for 30 minutes until a yellow/white turve solution is formed; (3) – centrifugation at 10000 rpm during 10 minutes until the solution appears light yellow with a white precipitate in the bottom of the flask, indicating the removing of the chloride ions present in the initial serum; (0.1 M of Cl- present in the initial serum) (4) – Cormay® serum pre-treated is compatible for incubation in the anode side of the PEDOT/PB-DMFC with a 100x MES buffer dilution; (5)- example of a stabilization protocol using the diluted pre-treated serum in a PEDOT/PB-DMFC assembly containing the MIP sensor, evidencing a stable system after 4/5 incubations.
- **Figure S2.** Steps in the PEDOT/PB-DMFC development. (1) – the Nafion® electrolyte incorporation in the Whatman paper, (2) ink deposition (anode/cathode) in both sides of the electrolyte, (3) paraffin impermeabilization in the surrounding area of the electrodes (50/50 paraffin/IPA), and (4) electrical connector's integration on the top edges with silver ink.; (5) this final assembly is isolated with glue tape to avoid leaking of the methanol solution and avoid short circuit; (6) the final PEDOT/PB-DMFC operates with a solution of methanol (0.5 M – 1 M) which is adsorbed in a square of adsorbent paper and with the cathode exposed to

air; (7) a typical polarization and power curves after signal activation and stabilization; PEDOT/PB-DMFC contains a MIP sensor for L1CAM detection.

- **Figure S3.** Example of the polarization curve calculations to obtain the normalized current density and power, highlighting the maximum power of each system, used for comparison of all the developed systems.
- **Figure S4.** Steady-state cyclic voltammograms obtained in a 3-electrode system in a PBS buffer solution (the 5th cycle is presented, −0.3 V to 1.2 V, 50 mV/s), for the assembly of the PEDOT sensing polymer on the anode layer of PEDOT/PB-DMFC-MIP in comparison with the control PEDOT/PB-DMFC-NIP and corresponding removal step in sulfuric acid solution.
- **Figure S5.** SEM analysis of a PEDOT/PB-DMFC/MIP and PEDOT/PB-DMFC/NIP electrodes, before and after activation and stabilization, evidencing some roughness modifications.
- **Figure S6.** Power curves were obtained in consecutive electrochemical readings using independent PEDOT/PB-DMFCs assemblies after stabilization and prior incubation with MES buffer (A), pre-treated diluted (100x) Cormay® serum (B) using 0.5 M MeOH solution for the readings.
- **Figure S7.** Photographs of the PEDOT/PB-DMFC-stack; the electrical set-up (A), the anode sensing side(B), and connected to the cathodes (C).

3. Supplementary Tables

- **Table S1.** Electrochemical data and normalized power calculation of the results presented in Figure 3 A-D.
- **Table S2.** Electrochemical data and power calculation of the results presented in Figure 5-A/B, D/E.

- **Table S3.** RGB coordinates determined at three different locations with the blue coordinate, and calculation of the average values and standard deviation.

1. EXPERIMENTAL SECTION

1.1. Reagents and solutions.

All the reagents were of analytical grade and the water was an ultrapure Milli-Q. A solution of Nafion® [20 wt%, alcohol based (propanol/ethanol) Quintech] was applied to a ≈ 2.2 cm^2 square paper strip (Whatman grade 40 filter papers) and covered on both sides with carbon based inks consisting of: Carbon Black with PtRu (40:20 wt%) catalyst (C/PtRu) on Vulcan XC-72R (Quintech) mixed with oxidized Multi Walled Carbon Nanotubes (MWCNT-COOH) (Sigma Aldrich) for the anode side, and Carbon Black with Pt (10 wt%) catalyst (C/Pt) on Vulcan XC-72R (Quintech) for the cathode side. The loading of the Pt catalyst in the anode layer was set as 2 mg/cm^2 and 0.5 mg/cm^2 for the cathode electrode. The inks were prepared using 2-propanol (IPA) (99.8%, Panreac) as solvent and Nafion® (20 wt%.) as binder using a microtip ultrasonic system. A solution of 0.01 M EDOT (97%, Alfa Aesar) in IPA/H_2O (4/1) was added to the anode ink and sonicated in ultrasonic bath for 60 s. Titanium (IV) oxide (anatase, Sigma Aldrich) was also added to the formulation for evaluating TiO_2 applicability as conductive component in ink. Different amounts of MWCNT-COOH were also tested in the ink formulation.

A paraffin solution (Sigma Aldrich) was diluted in 2-propanol (50/50) and applied around the active area of the PB-DMFC assembly (on both sides of the paper). It hydrophobized and strengthens the paper substrate. Conductive silver ink (RS Pro) was applied to the top edges for electrical connection (each side with an independent terminal, insulated with tape on the non-electrical side to avoid short circuits). Two buffer media were used in this work: 10 mM MES buffer (pH 6.0) obtained by dissolving 2-morpholinoethanesulfonic acid buffer (AppliChem) and PBS buffer with (pH 7.2) obtained by dissolving phosphate-buffered saline (Amresco). A recombinant human L1CAM protein (His-tag) (MyBiosource) stock solution was prepared by diluting the commercial reagent in buffer; less concentrated standards were prepared by accurately diluting the stock solution in the range of $1.0 \times 10^{-12} – 1.0 \times 10^{-8}$ M.

The polymerization solution consisted of 0.01 M EDOT (97%, Alfa Aesar) prepared in PBS (25 mL solution for each electrode), and L1CAM protein was used as a template during polymerization. Template

removal was performed electrochemically in 0.5 M H_2SO_4 (Sigma Aldrich). Electrochemical measurements were performed using an aqueous methanol solution (Honeywell) of 0.5 M for calibrations and 1.0 M for initial stabilization of the fuel cell. A pretreated human serum (Cormay® HN, PZ Cormay S.A) was used for the selectivity studies. The pretreatment consisted of adding silver nitrate ($AgNO_3$, 0.1 M) to the pure serum, to reduce the effect of chloride ions (0.1 M Cl^-). In this procedure, 10 mg of $AgNO_3$ was added to 1 mL of pure serum and left in a stir plate for 30 min. The mixture was then centrifugated at 10 000 rpm for 10 min, and a solid white precipitate (AgCl) was deposited at the bottom of the flask (Figure S1). This treated serum was 100x diluted in MES buffer and spiked with selected target concentrations. This medium was spiked with the same biomarker concentrations that were prepared in the buffer.

For the selectivity tests, L1CAM standard solutions were prepared in the presence of interfering species. These were carcinoembryonic antigen CEA (30 ng/mL), D-glucose (1.2 mg/mL; Alfa Aesar), and urea (1 mg/mL; Fagron) prepared in the buffer.

The paper supported EC was developed on top of baking paper, modified with polydimethylsiloxane (PDMS commercial kit SYLGARD™). After curing the PDMS, a promotor for gold adhesion, 3-Mercaptopropylmethyldimethoxysilane, MPTMS (Sigma Aldrich), was spin-coated on the surface of the paper/PDMS. A commercial dispersion of poly(2,3-dihydrothieno-1,4-dioxin)-poly(styrenesulfonate) (PEDOT:PSS) (Sigma Aldrich) was diluted in dimethyl sulfoxide (DMSO, 95/5(%)) and spread in the middle ring of the EC. 0.1 M solution of lithium perchlorate (Sigma Aldrich®) was used as the electrolyte in the EC paper device.

1.2. Equipment and apparatus

Electrochemical measurements were performed at lab-ambient conditions (21±2 ^oC) using a Methrom Autolab potentiostat/galvanostat with a PGSTAT302N, controlled by Nova software (NOVA 2.1). The certified current resolution of the PGSTAT302N is $30{\cdot}10^{-15}$ A; fairly enough for reliable tracking of the output current and power of the produced devices ranging in several μA and μW, respectively. The electrochemical

characterization and performance evaluation of the different PEDOT/PB-DMFCs were assessed using Sampled DC technique in the form of polarization curves, until OCV, current and power stabilization (single cell assembly). First, the PEDOT/PB-DMFC devices were activated by running several polarization curves using 1 M MeOH solution; polarization cycles were applied until a stable response of the cell (deviation of the obtained power $\leq$ 1%). A solution of 0.5 M MeOH was used for the calibration procedures. After each measurement, the PEDOT/PB-DMFC was washed with ultrapure water and dried with nitrogen to remove the unreacted methanol solution. Electrochemical impedance spectroscopy (EIS) was used to evaluate the electrical resistance of the PEDOT/carbon matrix/catalyst interface in OCV mode.

All electrochemical measurements recorded in the different experiments were normalized with respect to the area of the electrodes (2.2 cm^2). To follow and compare the electrochemical behaviour of the PEDOT/PB-DMFC assemblies, the maximum peak power was calculated. The calculated power values were normalized by the corresponding blank signal ($power_{sample}/power_{blank}$) to allow a better comparison between the different calibration experiments.

The anode and cathode nanoinks were performed using a Sonic Dismembrator (Fisherbrand™), with an operation frequency of 40 KHz, coupled to a 3 mm microtip. The PEDOT/PB-DMFC, PEDOT/PB-DMFC/MIP, and PEDOT/PB-DMFC/NIP anode layers were characterized by Raman spectroscopy, with a Thermo Scientific DXR Raman spectroscope equipped with a confocal microscope. Spectra were recorded with a 532 nm excitation laser in the range of 50 to 3500 cm^{-1} at a power of 7 mW with a 50× objective. The confocal aperture was set to 50 µm pinhole. The ultrasonically polymerized PEDOT/CB-PtRu powder was analysed with FTIR/ATR spectra in a Thermo Scientific iS10 in the range of 550 to 4000 cm^{-1} with a germanium (Ge) crystal at 250 scans. For comparison, spectra of the unmodified commercial CB-PtRu and EDOT monomer were also analysed (all solid samples were first dried in an oven at 37 ˚C before reading). Thermogravimetric analysis (TG) was used to follow the different steps of the anode ink nanocomposite formulation, in an inert atmosphere (nitrogen, 20 mL min^{-1}, heating rate 10 °C/min, from 30 to 800 ˚C), using

a Hitachi (TG/DTA7200) instrument. The morphology of the PEDOT/PB-DMFCs was studied using a scanning electron microscope (SEM) with cross-section and microstructure analysis.

1.3. Electrochemical and electrochromic assays

The electrochemical performance of the PEDOT/PB-DMFCs was evaluated using the typical polarization curves used to characterize fuel cell systems (obtained by Sampled DC technique). Calibrations of the PEDOT/PB-DMFCs/biosensor and the PEDOT/PB-DMFCs/NIP were performed by incubating L1CAM standard solutions in the range of 1.0×10^{-12} – 1.0×10^{-8} M in MES buffer. Prior to this, blank stabilization was performed by incubating the MES buffer on the modified anode until electrical signal stabilization was reached. During calibration, each solution (100 µL) was incubated on the anode surface for 20 minutes, starting with the lowest concentration. The system was covered with a glass square to efficiently distribute the solution over the entire active area and avoid evaporation. After each incubation and electrochemical measurement, the anode sensor area was carefully washed with ultrapure water and dried with nitrogen. This procedure was repeated for increasing L1CAM concentration standards prepared in MES buffer and diluted Cormay® serum (100 ×) to evaluate the ability of the PEDOT/PB-DMFC/biosensor to discriminate L1CAM. For the selectivity assays, 1.0×10^{-10} M L1CAM concentration was selected and incubated directly in the PEDOT/PB-DMFCs/MIP and the signal was measured. The same L1CAM concentration was doped with the different selected interferents and later incubated in the sensor area using different PEDOT/PB-DMFC assemblies (L1CAM standard + interferent for PEDOT/PB-DMFC/biosensor unit).

Calibrations with the hybrid PEDOT/PB-DMFC/biosensor stack connected to the EC were performed in buffer after the hybrid stack stabilized to the maximum voltage (≈1.5 V). The anode region of the different PEDOT/PB-DMFCs electrically connected to each other was incubated with the same concentration standards of L1CAM used in the single-cell calibrations. After washing the sensing anode with ultrapure water, methanol was added to the fuel cells anodes. After 5 minutes of stabilization, a 48 Mp smartphone camera with a resolution of 8000 x 6000 pixels (Samsung Galaxy A51) was used to take a photo of the EC

center ring and the color coordinates of these images were analyzed using ImageJ software (blue coordinate selection). A typical smartphone, with a similar medium resolution can be used to reproduce the data.

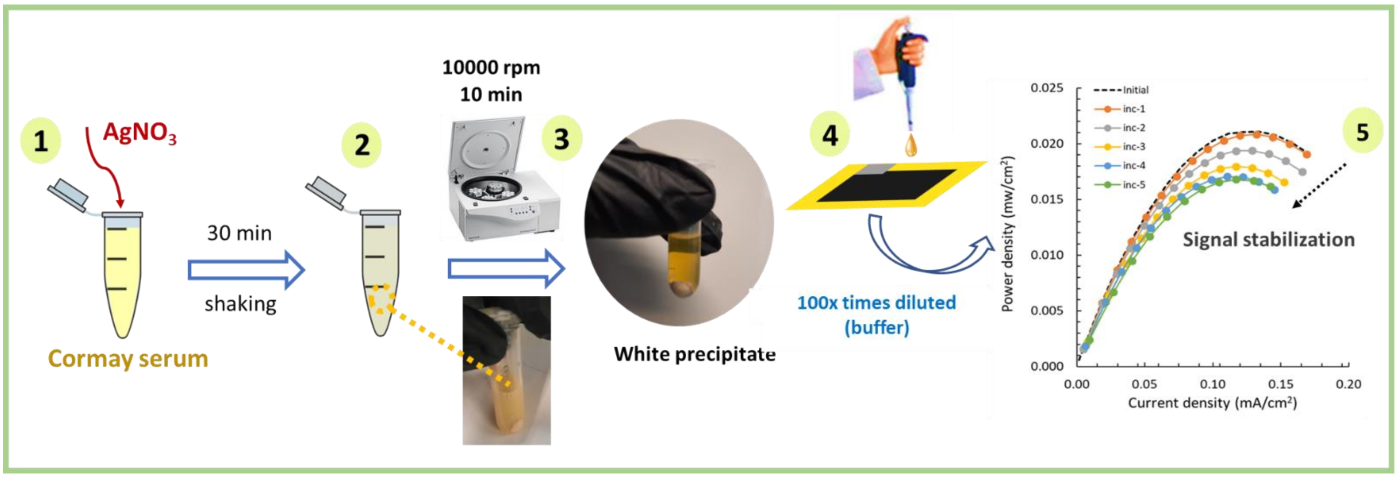

Figure S1 – Pre-treatment steps of the human serum (Cormay®) for L1CAM standards preparation: (1) – add $AgNO_3$ to the pure Cormay® serum; (2)- stir in a stirring plate for 30 minutes until a yellow/white turve solution is formed; (3) – centrifugation at 10000 rpm during 10 minutes until the solution appears light yellow with a white precipitate in the bottom of the flask, indicating the removing of the chloride ions present in the initial serum; (0.1 M of Cl^- present in the initial serum) (4) – Cormay® serum pre-treated is compatible for incubation in the anode side of the PEDOT/PB-DMFC with a 100x MES buffer dilution; (5)- example of a stabilization protocol using the diluted pre-treated serum in a PEDOT/PB-DMFC assembly containing the MIP sensor, evidencing a stable system after 4/5 incubations.

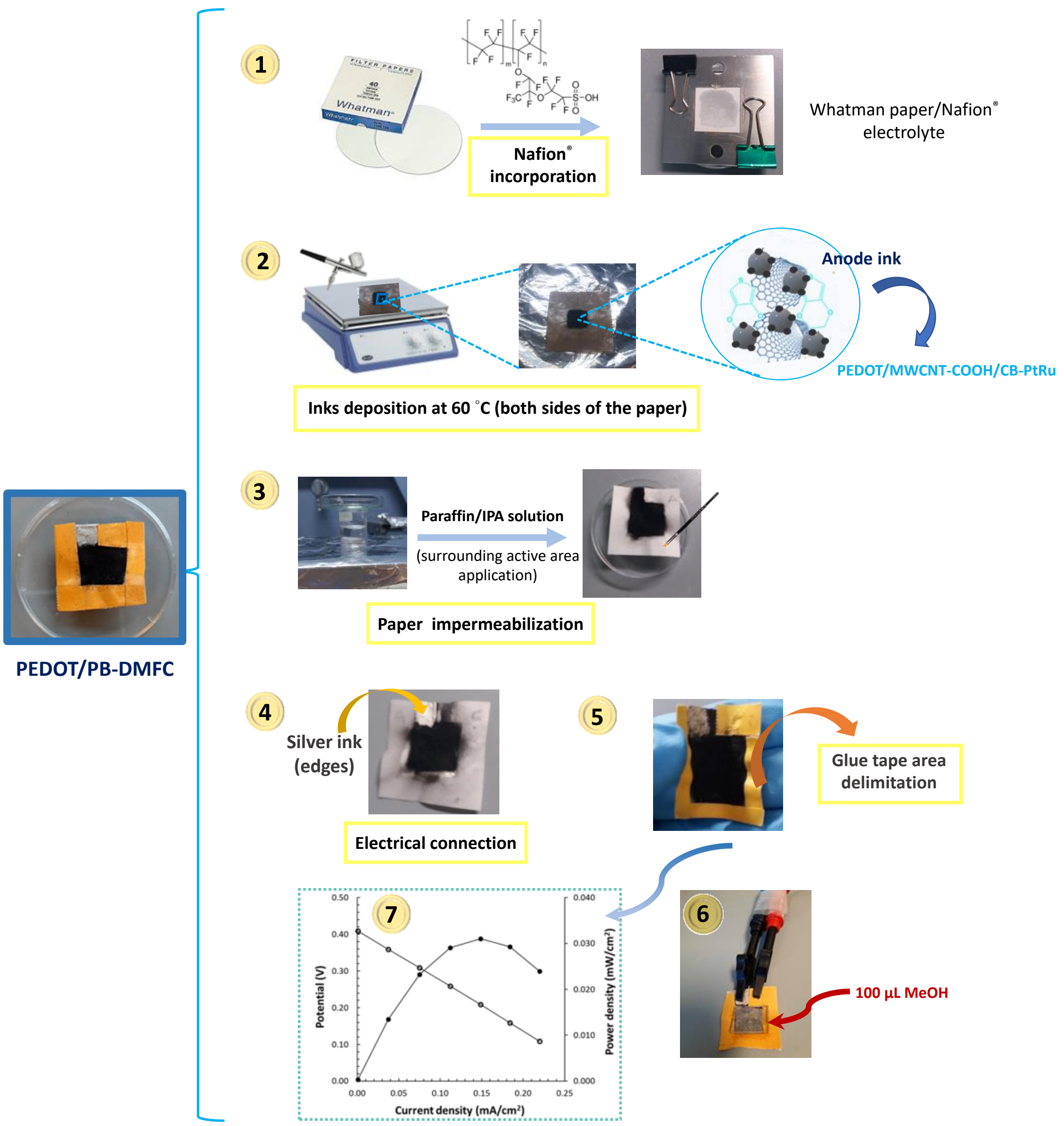

Figure S2 – Steps in the PEDOT/PB-DMFC development. (1) – the Nafion® electrolyte incorporation in the Whatman paper, (2) ink deposition (anode/cathode) in both sides of the electrolyte, (3) paraffin impermeabilization in the surrounding area of the electrodes (50/50 paraffin/IPA), and (4) electrical connector's integration on the top edges with silver ink.; (5) this final assembly is isolated with glue tape to avoid leaking of the methanol solution and avoid short circuit; (6) the final PEDOT/PB-DMFC operates with a solution of methanol (0.5 M – 1 M) which is adsorbed in a square of adsorbent paper and with the cathode exposed to air; (7) a typical polarization and power curves after signal activation and stabilization; PEDOT/PB-DMFC contains a MIP sensor for L1CAM detection.

Paraffin			
Potential/V	Current/A	*1Current density (μA/cm²)	*2Power (μW/cm²)
0.405121	-4.46E-07	0.2228	0.0903
0.380096	-2.63E-05	13.1577	5.0012
0.355072	-5.18E-05	25.9247	9.2051
0.330048	-7.83E-05	39.1693	12.9277
0.305023	-0.000105682	52.8410	16.1177
0.279999	-0.000133789	66.8945	18.7304
0.254974	-0.000162659	81.3295	20.7369
0.22995	-0.000192413	96.2065	22.1227
0.204926	-0.000222992	111.4960	22.8484
0.179901	-0.000254242	127.1210	22.8692
0.154877	-0.00028595	142.9750	22.1435
0.129852	-0.000318665	159.3325	20.6896
0.104828	-0.000351959	175.9795	18.4476

PTFE (50 μL)			
Potential/V	Current/A	*1Current density (μA/cm²)	*2Power (μW/cm²)
0.458984	-3.31E-06	1.6571	0.7606
0.43396	-3.12E-05	15.5991	6.7694
0.408936	-5.58E-05	27.8778	11.4002
0.383911	-7.85E-05	39.2609	15.0727
0.358887	-0.000100098	50.0490	17.9619
0.333862	-0.000120941	60.4705	20.1888
0.308838	-0.000141663	70.8315	21.8755
0.283813	-0.000161774	80.8870	22.9568
0.258789	-0.000182495	91.2475	23.6138
0.233765	-0.00020401	102.0050	23.8452
0.20874	-0.000225739	112.8695	23.5604
0.183716	-0.000248291	124.1455	22.8075
0.158691	-0.000271698	135.8490	21.5580
0.133667	-0.000295593	147.7965	19.7555
0.108643	-0.000319977	159.9885	17.3816

*1 Current density = Current (μA)/ PB-DMFC area (2 cm²)

*2 Power = Current density x Potential

Figure S3 – Example of the polarization curve calculations to obtain the normalized *current density* and *power*, highlining the maximum power of each system, used for comparison of all the developed systems.

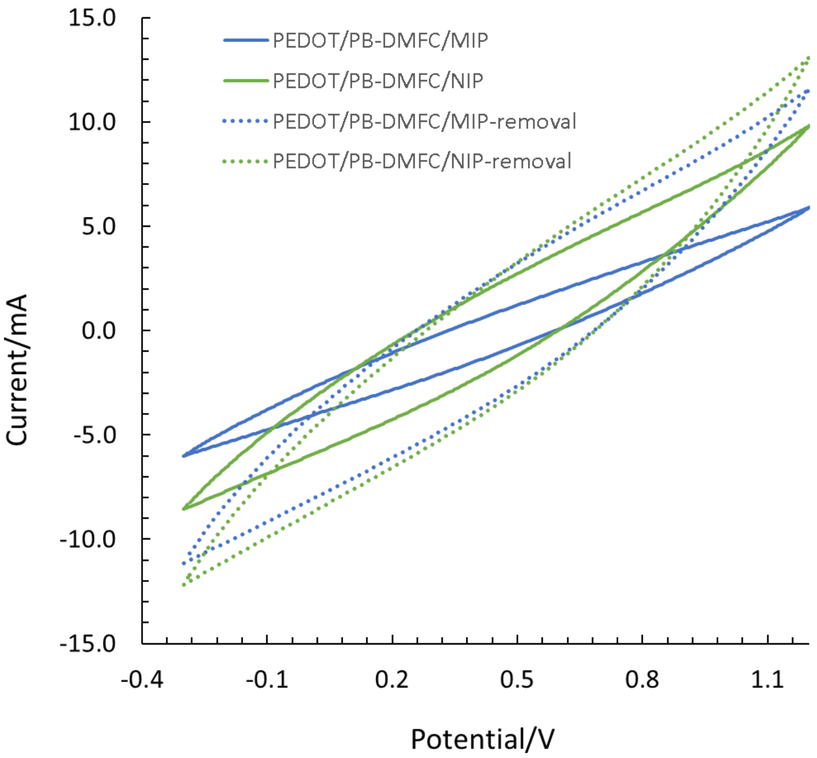

Figure S4 – Steady state cyclic voltammograms obtained in a 3-electrode system in a PBS buffer solution (the 5th cycle is presented, −0.3 V to 1.2 V, 50 mV/s), for the assembly of the PEDOT sensing polymer on the anode layer of PEDOT/PB-DMFC-MIP in comparison with the control PEDOT/PB-DMFC-NIP and corresponding removal step in sulfuric acid solution.

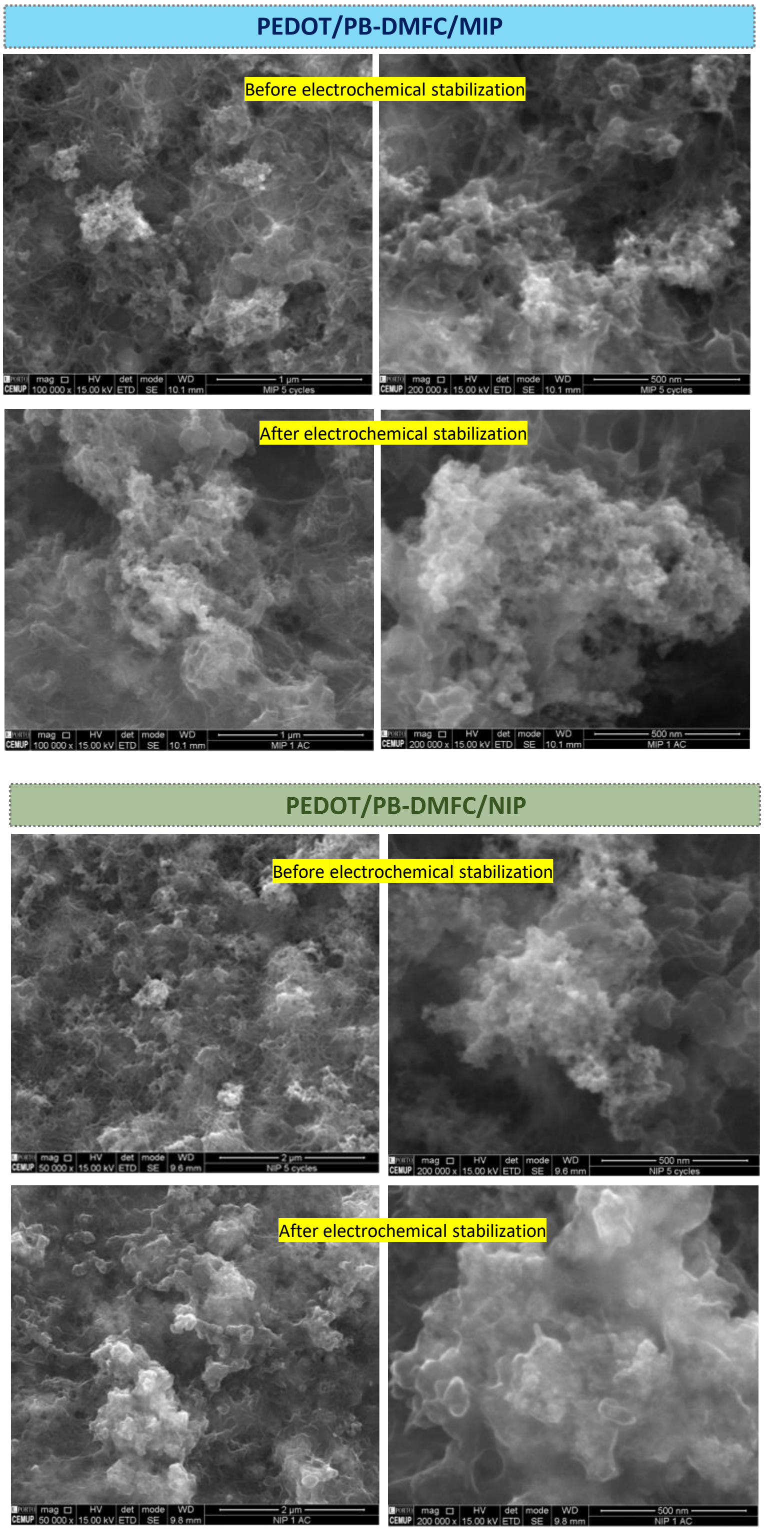

Figure S5 – SEM analysis of a PEDOT/PB-DMFC/MIP and PEDOT/PB-DMFC/NIP electrodes, before and after activation and stabilization, evidencing some roughness modifications.

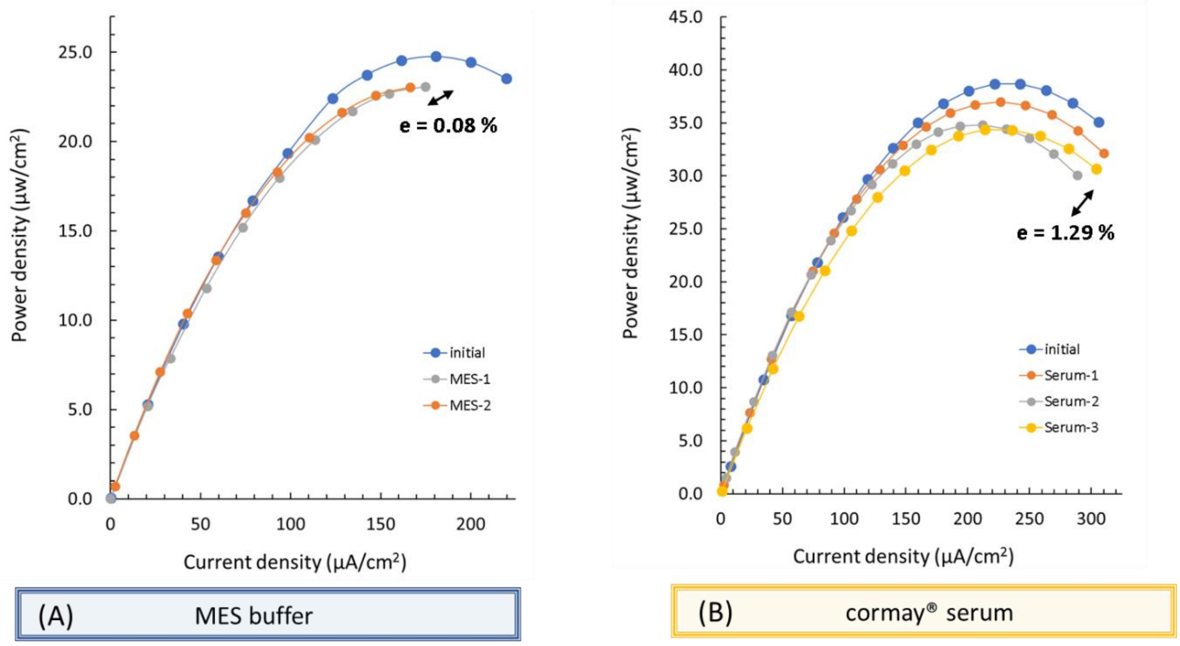

Figure S6 - Power curves obtained in consecutive electrochemical readings using independent PEDOT/PB-DMFCs assemblies after stabilization and prior incubation with MES buffer (A), pre-treated diluted (100x) Cormay® serum (B) using 0.5 M MeOH solution for the readings.

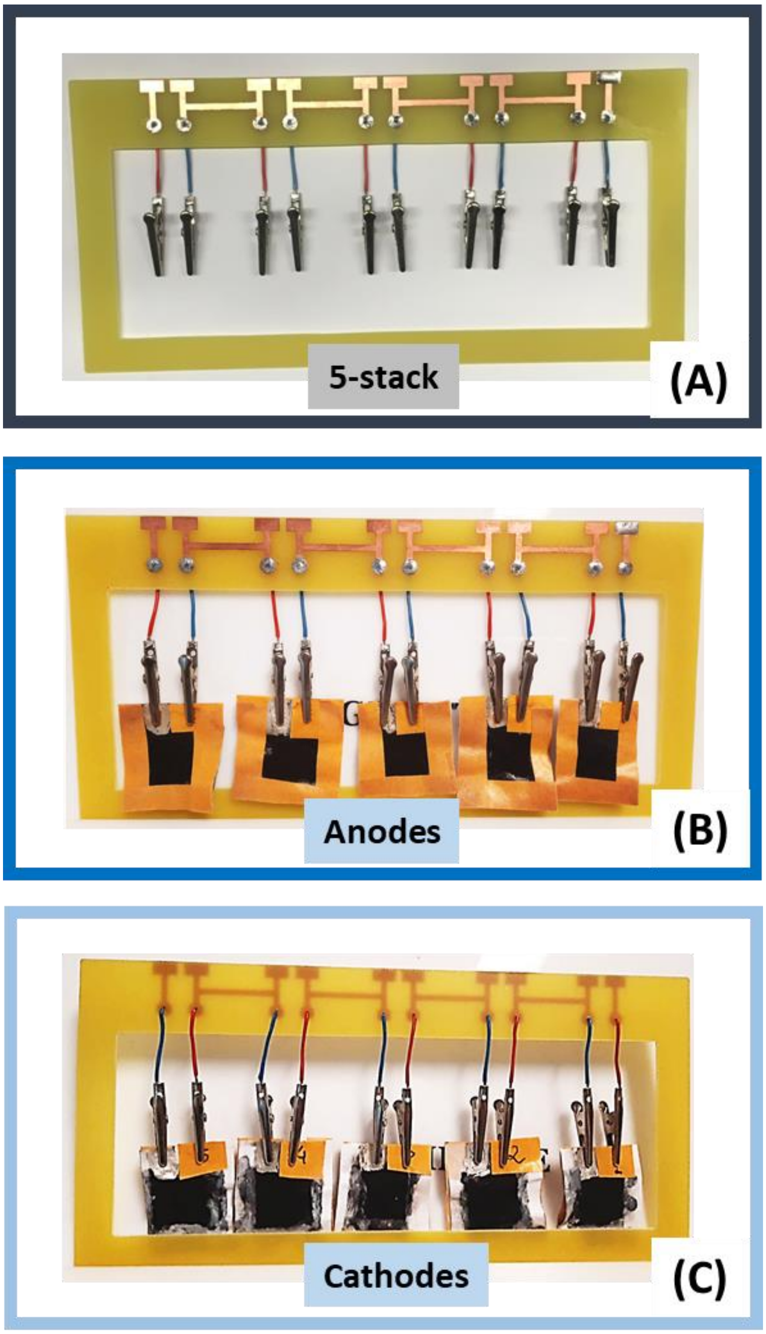

Figure S7 – Photographs of the PEDOT/PB-DMFC-stack; the electrical set-up (A), the anode sensing side(B), and connected to the cathodes (C).

Table S1. Electrochemical data and power calculation of the results presented in Figure 3 A-D.

Sample	Potential/V	Current density (μA/cm^2)	Power (μW/cm^2)
Paraffin	0.18	127.12	22.87
PTFE (50 μL)	0.23	102.01	23.85
PTFE (25 μL)	0.17	100.62	17.47
0.005 M EDOT	0.16	85.57	13.79
0.01 M EDOT	0.14	60.20	8.48
0.02 M EDOT	0.21	21.70	4.56
EDOT + 5mg TiO_2	0.22	138.46	29.92
EDOT + 10 mg TiO_2	0.13	89.57	11.96
EDOT + 1 mg MWCNTs-COOH	0.19	150.67	29.31
EDOT + 3 mg MWCNTs-COOH	0.16	345.46	56.82
EDOT + 5 mg MWCNTs-COOH	0.18	418.24	75.88

Table S2. Electrochemical data and power calculation of the results presented in Figure 5-A/B, D/E.

PEDOT/PB-DMFC/biosensor			
**Buffer calibration**	**Potential/V**	**Current density ($\mu A/cm^2$)**	***[1]Power ($\mu W/cm^2$)**
1 x 10 $^{-12}$ M L1CAM	0.14	95.91	13.25
1 x 10 $^{-11}$ M L1CAM	0.14	83.69	11.93
1 x 10 $^{-10}$ M L1CAM	0.13	79.13	10.31
1 x 10 $^{-9}$ M L1CAM	0.11	66.24	7.27
1 x 10 $^{-8}$ M L1CAM	0.11	46.46	5.05
**Cormay serum calibration**	**Potential/V**	**Current density ($\mu A/cm^2$)**	***[1]Power ($\mu W/cm^2$)**
1 x 10 $^{-12}$ M L1CAM	0.14	225.64	31.64
1 x 10 $^{-11}$ M L1CAM	0.13	213.10	28.26
1 x 10 $^{-10}$ M L1CAM	0.13	192.49	24.55
1 x 10 $^{-9}$ M L1CAM	0.13	147.51	19.74
1 x 10 $^{-8}$ M L1CAM	0.11	125.89	13.91

*[1] Average power = $Power_{standard}/Power_{blank}$

Table S3 RGB coordinates determined at three different locations with the blue coordinate, and calculation of the average values and standard deviation.

	blue coordinate (RGB)				
**Sample**	**P1**	**P2**	**P3**	**average**	**SD**
initial	105.897	112.344	111.323	109.85	0.511
blank	110.878	117.608	116.55	115.01	0.529
1.00E-12	105.401	108.838	109.161	107.80	1.701
1.00E-11	103.579	100.388	102.861	102.28	1.674
1.00E-10	99.855	99.143	97.386	98.79	1.038
1.00E-09	95.796	93.612	95.344	94.92	0.941
1.00E-08	88.288	88.541	89.637	88.82	0.585
